# New biofunctional effects of oleanane-type triterpene saponins

**DOI:** 10.1007/s11418-023-01730-w

**Published:** 2023-07-12

**Authors:** Hisashi Matsuda, Toshio Morikawa, Seikou Nakamura, Osamu Muraoka, Masayuki Yoshikawa

**Affiliations:** 1 Department of Pharmacognosy, Kyoto Pharmaceutical University, Misasagi, Yamashina-Ku, Kyoto, 607-8412 Japan; 2 Pharmaceutical Research and Technology Institute, Kindai University, 3-4-1 Kowakae, Higashi-Osaka, Osaka, 577-8502 Japan

**Keywords:** Triterpene saponin, Biofunctional effect, Gastric emptying, Gastrointestinal transit, Gastric mucosal protection, Appetite suppression

## Abstract

In the current review, we describe the novel biofunctional effects of oleanane-type triterpene saponins, including elatosides, momordins, senegasaponins, camelliasaponins, and escins, obtained from *Aralia elata* (bark, root cortex, young shoot), *Kochia scoparia* (fruit), *Polygala senega* var. *latifolia* (roots), *Camellia japonica* (seeds), and *Aesculus hippocastanum* (seeds), considering the following biofunctional activities: (1) inhibitory effects on elevated levels of blood alcohol and glucose in alcohol and glucose-loaded rats, respectively, (2) inhibitory effects on gastric emptying in rats and mice, (3) accelerative effects on gastrointestinal transit in mice, and (4) protective effects against gastric mucosal lesions in rats. In addition, we describe (5) suppressive effects of the extract and chakasaponins from *Camellia sinensis* (flower buds) on obesity based on inhibition of food intake in mice. The active saponins were classified into the following three types: (1) olean-12-en-28-oic acid 3-*O*-monodesmoside, (2) olean-12-ene 3,28-*O*-acylated bisdesmoside, and (3) acylated polyhydroxyolean-12-ene 3-*O*-monodesmoside. Furthermore, common modes of action, such as involvements of capsaicin-sensitive nerves, endogenous NO and PGs, and possibly sympathetic nerves, as well as common structural requirements, were observed. Based on our findings, a common mechanism of action might mediate the pharmacological effects of active saponins. It should be noted that the gastrointestinal tract is an important action site of saponins, and the role of the saponins in the gastrointestinal tract should be carefully considered.

## Introduction

Most saponins, characterized by triterpenes or steroids with oligoglycoside linkage, are well-known to exert soap-like foaming, fish toxicity, and hemolysis, however herbs with high saponin content have been used as traditional medicines, dietary supplements, and development of cosmetics and drugs. Recently, the development of isolation techniques such as preparative HPLC, along with instruments for elucidating chemical structures such as high-resolution NMR, MS, and X-ray analyses, have facilitated the isolation and elucidation of chemical structures of saponins in a short period and from minimal quantities. Therefore, the chemical structures of numerous saponins have been elucidated. However, their biofunctional effects and the mechanism of actions remain poorly explored, except for saponins contained in several important natural medicines such as glycyrrhiza [1], ginseng [2], and bupleurum root [3].

We have previously elucidated the active constituents of natural medicines considering the aspects of chemistry and pharmacology, revealing activities against allergy and inflammation, diabetes, obesity, and proliferation and metastasis of tumor cells. During our studies, we explored a large number of triterpene saponins, comprising triterpene as a sapogenol, from various natural medicines and medicinal foods and identified interesting biofunctional effects.

In the present review, we summarize the oleanan-type triterpene saponins isolated from *Aralia elata* Seem. (bark, root cortex, young shoot), *Kochia scoparia* (L.) Schrad. [*Bassia scoparia* (L.) A.J. Scott (fruit)], *Polygala senega* L. var. *latifolia* Torrey *et* Gray (roots) (senega), *Camellia japonica* L. (seeds), and *Aesculus hippocastanum* L. (seeds)(horse chestnut seed), with suppressive or delayed effects on elevated blood ethanol and glucose levels in rats and gastric emptying (GE) in rats and mice, accelerative effects on gastrointestinal transit (GIT) in mice, protective effects on gastric mucosal lesions in rats, and anti-inflammatory activity in mice. Furthermore, we describe the anti-obesity and suppressive effects of food intake mediated by an extract and chakasaponins from *Camellia sinensis* (L.) O. Kuntze (flower buds) (tea-flower) in mice [4].

## Effects on increased blood ethanol levels after ethanol loading in rats

Alcoholism is a major health problem globally and has been associated with considerable physiological and social challenges. Chug-a-lugging of alcoholic drinks (‘Ikkinomi’ in Japanese) can induce acute alcohol toxicity with acidosis, heart failure, and respiratory depression caused by autonomic nerve and cerebrum dysfunction. Long-term alcohol consumption in large quantities can induce numerous disorders, such as hepatopathy, gastrointestinal disorders, chronic pancreatitis, peripheral nerve disorder, and hypertension. Therefore, inhibitors of alcohol absorption may exert potential preventive effects against acute and chronic alcoholism.

To identify compounds that could suppress elevated blood ethanol levels, we performed ethanol loading and explored the potential of saponins from various natural medicines, including those traditionally employed for detoxification against ethanol poisoning. For screening, the test samples were orally administered to fasted rats. After 1 h, 20% aqueous ethanol (5 mL/kg) was orally (p.o.) or intraperitoneally (i.p.) administered, and blood samples were collected at 1, 2, and 3 h after ethanol loading, followed by detection of blood ethanol levels. Saponin fractions of *A. elata* (bark root cortex and young shoot), *K. scoparia* (fruit), senega, *C. japonica* (seeds), and horse chestnut seeds were found to suppress the elevated blood ethanol levels in ethanol-loaded rats.

**Fig. 1:**
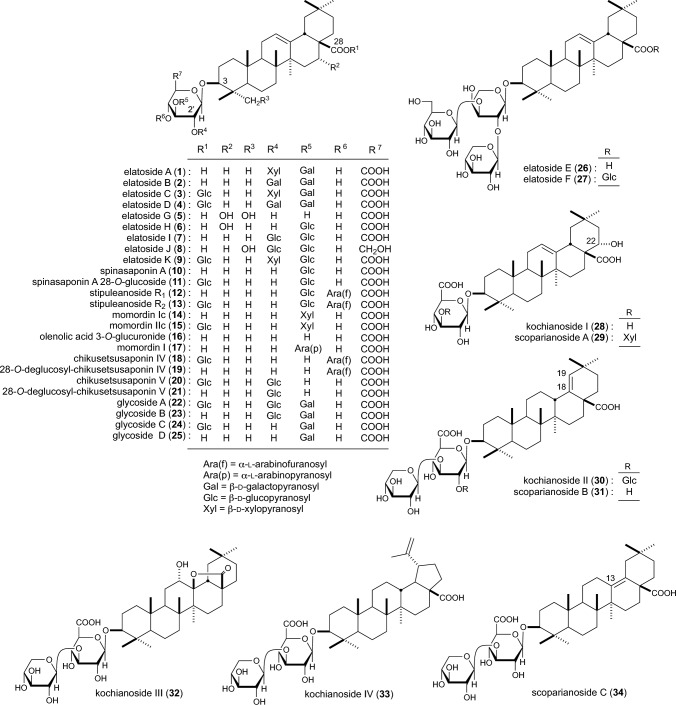
Chemical structures of elatosides A–D (**1**–**4**), E (**26**), F (**27**), and G–K (**5**–**9**), spiasaponin A (**10**) and its 28-*O*-glucoside (**11**), and stipuleanosides R_1_ (**12**) and R_2_ (**13**) from *A. elata*, momordins Ic (**14**) and IIc (**15**), kochianosies I–IV (**28**, **30**, **32**, **33**), and scoparinosides A–C (**29**, **31**, **34**) from *K. scoparia*, and related glycosides (**16**–**25**)

Using bioassay-guided separation, 11 new saponins from *A. elata* (bark, root cortex, and young shoot) [elatosides A–D (**1**–**4**), E (**26**), F (**27**), G–K (**5**–**9**)], along with known saponins [e.g., spinasaponin A (**10**) and its 28-*O*-β-d-glucopyranoside (**11**), stipuleanosides R_1_ (**12**) and R_2_ (**13**) [5–8], and 7 new saponins [kochianosides I (**28**), II (**30**), III (**32**), and IV (**33**) and scoparianosides A (**29**), B (**31**), and C (**34**)] and known saponins [e.g., momordins Ic (**14**) and IIc (**15**)] were isolated from the fruit of *K. scoparia* [9, 10] (Fig. 1). The saponins obtained in a sufficient amount from natural medicines were subjected to bioassays.

Oleanolic acid 3-*O*-monodesmosides [elatosides A (**1**) and B (**2**), spinasaponin A (**10**), and stipuleanoside R_1_ (**12**)] exhibited potent inhibitory effects on elevated blood ethanol levels at a dose of 100 mg/kg (p.o.), affording an inhibition of 86.0–100% at 1 h. Particularly, **1** and **10** inhibited elevated blood ethanol levels dose-dependently at 25–100 mg/kg (inhibition: 54.4–100% at 1 h). However, oleanolic acid 3,28-*O*-bisdesmosides [elatosides C (**3**) and D (**4**), spinasaponin A 28-*O*-glucoside (**11**), stipuleanoside R_2_ (**13**)] exerted a weak or no effect. Accordingly, the oleanolic acid 3-*O*-monodesmoside structure is essential for activity [5].

Momordin Ic (**14**, 25–100 mg/kg) potently inhibited elevated ethanol levels (64.5–80.5% at 1 h). In addition, its 2′-*O*-β-d-glucopyranoside (100 mg/kg) inhibited elevated ethanol levels, but its action was weaker than that of **14** (60.0% at 1 h); momordin Ilc (**15**) and its 2′-*O*-β-d-glucopyranoside failed to exert this action [10]. Furthermore, to confirm the structural requirement, we examined the inhibitory effects of oleanolic acid, oleanolic acid 3-*O*-monodesmosides [oleanolic acid 3-*O*-glucuronide (**16**), 28-*O*-deglucosyl-chikusetsusaponins IV (**19**) and V (**21**)], and oleanolic acid 3,28-*O*-bisdesmosides [chikusetsusaponins IV (**18**) and V (**20**)] from several natural medicines on elevated ethanol levels. Compounds **16** and **19** (25–100 mg/kg) exhibited potent inhibitory activity (65.6–100% at 1 h) similar to **1** and **2**, while **21** (50, 100 mg/kg) induced less inhibition (18.0 and 90.2% at 1 h) than **19**. These findings imply that the 2′-*O*-β-d-glucopyranosyl group reduced activity. Compounds **18** and **20** with 3,28-*O*-bisdesmoside structure lacked activity, and the common sapogenol, oleanolic acid, also showed poor activity [5].

Following i.p. administration of 20% aqueous ethanol to rats, **16** failed to decrease blood ethanol levels. Although the inhibitory mechanism of oleanolic acid 3-*O*-monodesmosides on elevated blood levels remains poorly understood, we speculated that these monodesmosides decrease the blood ethanol concentration by suppressing absorption across the cell membranes of the digestive tract or delaying absorption by inhibition of GE, as described in the section of the mode of action, but not by acceleration of ethanol metabolism. Based on the pharmacological assessments, these oleanolic acid 3-*O*-monodesmosides exerted more potent inhibitory activity than the olean-12-ene 3,28-*O*-acylated bisdesmosides (senegins and senegasaponins) and the acylated polyhydroxyolean-12-ene 3-*O*-monodesmosides (camelliasaponins and escins), as described in the following sections.

**Fig. 2:**
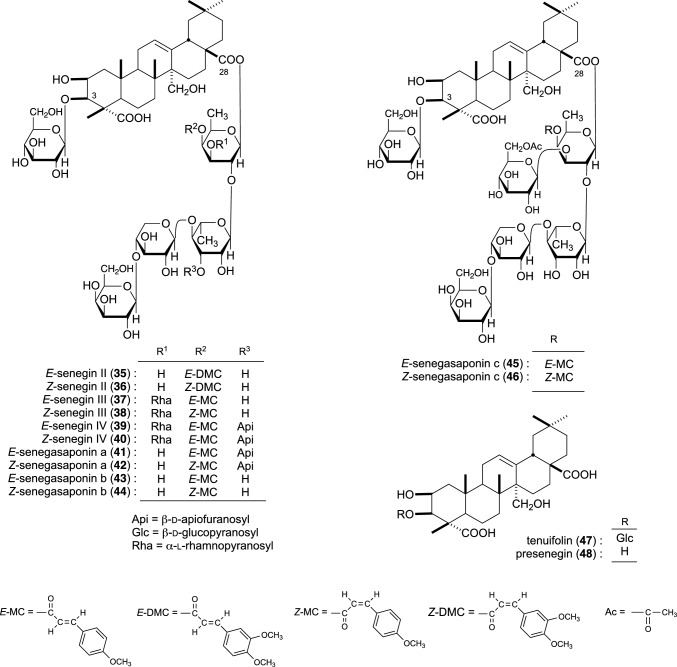
Chemical structures of *E*,*Z*-senegins II–IV (**35**–**40**) and *E*,*Z*-senegasaponins a–c (**41**–**46**) from senega, and tenuifolin (**47**) and presenegin (**48**)

Nine new saponins, *Z*-senegins II (**36**), III (**38**), and IV (**40**) and *E*,*Z*-senegasaponins a (**41**, **42**), b (**43**, **44**), and c (**45**, **46**) were isolated from the saponin fraction of senega capable of suppressing elevated blood ethanol levels (Fig. 2). Given that the geometrical isomeric structures of the 4-methoxycinnamoyl group in each senegasaponin show tautomer-like behavior in acidic aqueous solution, *E-* and *Z*-mixtures of senegins and senegasaponins were used for bioassays. The inhibitory effects of *E,Z*-senegins II (mixture of **35** and **36**), *E,Z*-senegasaponins a (**41** and **42**) and b (**43** and **44**), and desacylsenegasaponins a and b on elevated ethanol levels in rats were examined. *E*,*Z*-Senegins II (**35**, **36**) and *E*,*Z*-senegasaponins a (**41**, **42**) and b (**43**, **44**) potently inhibited elevated blood ethanol levels at 100 mg/kg, p.o. (91.3, 88.0, and 86.0% at 1 h). Desacylsenegasaponins a and b also tended to inhibit the elevated blood ethanol levels at 100 mg/kg, p.o. (56.0 and 48.0% at 1 h); however, their effects were weaker than those of *E,Z*-senegasaponins a (**41**, **42**) and b (**43**, **44**). Furthermore, the common prosapogenol and the genuine sapogenol of senegasaponins and senegins, tenuifolin (**47**) and presenegin (**48**), all lacked activity. *E*,*Z*-Senegins III (**37**, **38**) also showed some activity, however considerably weaker than that of *E*,*Z*-senegins II (**35**, **36**). *E*,*Z*-Senegins IV (**39**, **40**) and desacylsenegins III and IV were found to lack inhibitory activity [11, 12].

**Fig. 3:**
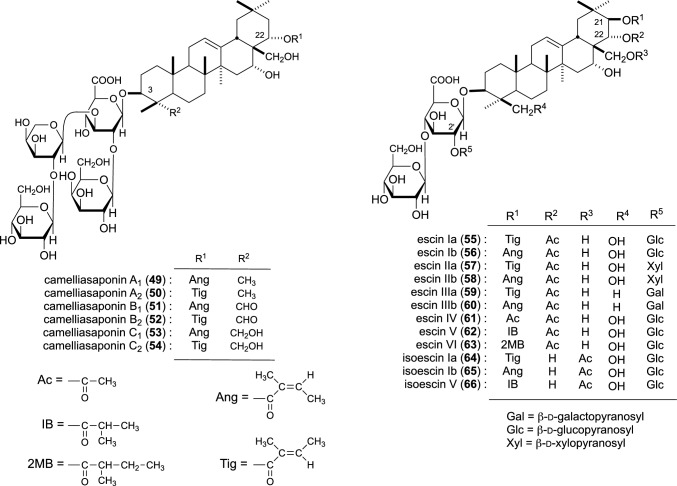
Chemical structures of camelliasaponins A_1_–C_2_ (**49**–**54**) from camellia seeds and escins Ia–VI (**55**–**63**) and isoescins Ia (**64**), Ib (**65**), and V (**66**) from horse chestnuts seeds

Six new saponins [camelliasaponin A_1_–C_2_ (**49**–**54**)] from camellia seeds (the seeds of *C. japonica*) [13] and 12 new saponins [escins Ia–VI (**55**–**63**), isoescins Ia (**64**), Ib (**65**), V (**66**)] from the seeds of horse chestnut tree (*A. hippocastanum*, ‘Seiyou-tochinoki’ in Japanese) were isolated [14, 15] (Fig. 3). Among the isolated camelliasaponins, the inhibitory effects of camelliasaponins B_1_ (**51**), B_2_ (**52**), C_1_ (**53**), and C_2_ (**54**) on elevated blood ethanol levels were examined. All camelliasaponins (**51**–**54**) exerted inhibitory effects at a dose of 100 mg/kg (24.6–82.5% at 1 h). Particularly, **51** exhibited the most potent inhibitory activity (82.5% at 1 h), and **52** exhibited the weakest inhibition (24.6% at 1 h). Conversely, desacyl-camelliasaponins B and C lacked activity [13].

Escins IIa (**57**) and IIb (**58**) exhibited inhibitory effects at doses of 50 and 100 mg/kg (31.5–86.2% at 1 h), while escins Ia (**55**) and lb (**56**) showed only weak activity at a dose of 100 mg/kg (7.4 and 20.4% at 1 h). Accordingly, it can be suggested that the 2′-*O*-β-d-xylopyranosyl group in **57** and **58** is essential to exert potent inhibitory activity, while the 2′-*O*-β-d-glucopyranosyl group could reduce activity. Desacylescins I and II lacked activity [14].

## Effects on elevated blood glucose levels after sugar loading in rats

The bark and root cortex of *A. elata* (‘Taranoki’ in Japanese) have been used for tonic, antiarthritic, and antidiabetic purposes in Chinese traditional medicines. It has been suggested that saponins isolated from this medicinal plant could reduce blood glucose levels. In the screening test, d-glucose (0.5 g/kg, p.o.) or sucrose (1 g/kg, p.o.) was orally loaded after administering the test sample in fasted rats. Blood samples were collected at 0.5, 1.0, and 2.0 h after sugar administration, and serum or plasma glucose levels were determined.

The effects of elatosides A (**1**), C (**3**), E (**26**), and F (**27**), and stipuleanosides R_1_ (**12**) and R_2_ (**13**) from the root cortex of *A. elata* and the other oleanolic acid glycosides [oleanolic acid 3-*O*-glucuronide (**16**), chikusetsusaponin IV (**18**)] were examined on elevated plasma glucose levels in oral sucrose-loaded rats. Oleanolic acid 3-*O*-monodesmosides (**l**, **12**, **16**, **26**) at a dose of 100 mg/kg (p.o.) inhibited the elevated plasma glucose levels (inhibition: 56.8–75.9% at 0.5 h), while 3,28-*O*-bisdesmosides with a 4′-*O*-α-l-arabinofuranosyl group (**13**, **18**) tended to inhibit the elevated plasma glucose levels (25.3 and 51.3% at 0.5 h); these effects were distinct from inhibitory effects of oleanolic acid glycosides on elevated ethanol levels described previously. Elatoside E (**26**) exerted the most potent activity (75.9% at 0.5 h), while oleanolic acid 3,28-*O*-bisdesmosides with a 2′,3′-*O*-diglycoside moiety (**3**, **27**) and oleanolic acid showed less activity [7]. Furthermore, the saponin fraction (200 mg/kg, p.o.) from the young shoot of *A. elata* inhibited the increased plasma glucose levels after loading of glucose in rats. Using bioassay-guided separation, five new saponins, i.e., elatosides G (**5**), H (**6**), I (**7**), J (**8**), and K (**9**), were isolated from the young shoot of *A. elata*. Elatosides G (**5**), H (**6**), and I (**7**) exhibited potent activity at a dose of 100 mg/kg (62.1–77.1% at 0.5 h). In contrast, the 3,28-*O*-bisdesmoside of oleanolic acid, elatoside K (**9**), showed less activity [6].

Moreover, momordins from the fruit of *K. scoparia* (‘Tonburi’ in Japanese), senegasaponins from senega, and escins from horse chestnut seeds exerted inhibitory effects on elevated serum glucose levels after glucose-loading in rats. The major saponins, momordin Ic (**14**) and its 2′-*O*-β-d-glucopyranoside, both of which possess the 28-carboxyl group and the 3-*O*-glucuronide moiety, showed potent inhibitory effects against elevated serum glucose levels in glucose-loaded rats at a dose of 100 mg/kg (p.o.), however **14** tended to exert stronger activity than its 2′-*O*-β-d-glucopyranoside (81.2 and 65.3% at 0.5 h). Conversely, the 3,28-*O*-bisdesmosides, momordin Ilc (**15**) and its 2′-*O*-β-d-glucopyranoside, lacked this activity [10].

Furthermore, we isolated 4 new triterpene saponins, calendasaponins A–D, and known principal saponins such as glycosides A–D (**22**–**25**) from the flowers of *Calendula officinalis* L. (so called ‘Marigold’) which has been used for inflammation of the oral and pharyngeal mucosa, wounds, and burns. Among the oleanolic acid 3-*O*-monodesmosides, glycoside D (**25**, 50 mg/kg) significantly inhibited elevated serum glucose levels in glucose-loaded rats (inhibition: 66.6% at 0.5 h), but glycoside B (**23**, 50 mg/kg) possessing the 2′-*O*-β-d-glucopyranosyl group did not. Two oleanolic acid 3,28-*O*-bisdesmosides, glycosides A (**22**) and C (**24**), also lacked the effect [16].

The inhibitory effects of *E*,*Z*-senegins II (**35**, **36**), III (**37**, **38**), and IV (**39**, **40**), *E*,*Z*-senegasaponins a (**41**, **42**), b (**43**, **44**), and c (**45**, **46**), and their desacyl derivatives were examined on elevated blood glucose levels in the oral glucose-loaded rats. *E*,*Z*-Senegins II (**35**, **36**) and III (**37**, **38**) inhibited the elevated plasma glucose levels at a dose of 100 mg/kg (43.3 and 32.1% at 0.5 h), while *E,Z*-senegins IV (**39**, **40**) had a weak effect. Under the same conditions, desacylsenegins III and IV lacked activity. *E*,*Z*-Senegasaponins a (**41**, **42**) and b (**43**, **44**) also exhibited inhibitory activity at a dose of 100 mg/kg (65.9 and 42.2% at 0.5 h). *E*,*Z*-senegasaponins c (**45**, **46**) showed weak inhibitory effects (14.8% at 0.5 h). Desacylsenegasaponin a also exerted inhibitory effects (27.8% at 0.5 h); however the effect was weaker than the corresponding *E*,*Z*-senegasaponins a (**41**, **42**). Furthermore, *E*,*Z*-senegasaponins a (**41**, **42**) and desacylsenegasaponin a showed more potent activity than *E*,*Z*-senegasaponins b (**43**, **44**) and desacylsenegasaponin b, respectively. It should be noted that the acyl group in senegasaponins and senegins is important for exerting potent activity [11, 12].

The major saponin constituents, escins Ia (**55**), lb (**56**), Ila (**57**), and Ilb (**58**) in the saponin fraction of horse chestnut seeds, could suppress elevated plasma glucose levels at a dose of 100 mg/kg (inhibition: 36.9–77.0% at 0.5 h), with escin IIa (**57**) exhibiting the most potent activity (77.0% at 0.5 h). In addition, escins IIa (**57**) and IIb (**58**), possessing the 2′-*O*-β-d-xylopyranosyl group in their oligosaccharide part, showed substantially more potent activity than escins Ia (**55**) and Ib (**56**), which possess the 2′-*O*-β-d-glucopyranosyl group. Conversely, desacylescins I and II lacked activity [14].

Furthermore, the fresh roots and leaves of sugar beet (*Beta vulgaris* L.) showed potent inhibitory effects on elevated serum glucose levels in glucose-loaded rats. We isolated 10 saponins, called betavulgarosides I–X, with novel dioxolane-type or acetal-type substituents, both of which are presumed to be biosynthesized through an oxidative degradation process of a terminal monosaccharide moiety, and their inhibitory effects on elevated plasma glucose levels have been reported in glucose-loaded rats [17–19]. Six new oleanane-type triterpene saponins (gymnemoside-a, -b, -c, -d, -e, -f) were isolated from the leaves of *Gymnema sylvestre* R. Br. Gymnemic acids III and V exhibited weak inhibitory effects on elevated serum glucose levels in glucose-loaded rats. Furthermore, the inhibitory effects of gymnemosides-c, -d, -e, and -f and principal triterpene glycosides (gymnemic acids I–V) were examined on d-[U-^14^C]glucose (2 mM) uptake in rat small intestinal fragments at 30℃ for 6 min. Several saponins, including gymnemic acids II and IV, as well as oleanolic acid 3-*O*-glucuronide (**16**) and escin Ia (**55**), inhibited glucose uptake at 0.5 mM [20, 21].

## Mode of action of inhibitory effects on elevated blood glucose levels

Next, the mode of action through which active saponins mediated their inhibitory effects on blood glucose elevation was examined using oleanolic acid 3-*O*-monodesmosides [momordin Ic (**14**), oleanolic acid 3-*O*-glucuronide (**16**)][22].

### Effects on blood glucose levels of normal rats, intraperitoneal glucose-loaded rats, and alloxan-induced diabetic mice

The regulation of serum glucose is controlled by several factors, such as the secretion and release of hormones (e.g*.*, insulin and glucagon), transport of sugar in the digestive tract, and glucose absorption via membranes of the small intestine.

Tolbutamide can increase insulin secretion to reduce serum glucose levels in normal and glucose-loaded rats. Both saponins (**14**, **16**) dose-dependently inhibited elevated serum glucose levels in oral glucose-loaded rats. However, **14** and **16** at 50 mg/kg did not decrease serum glucose levels in glucose-untreated (normal) rats nor serum glucose elevation in intraperitoneal glucose-loaded rats. Insulin (1 U/kg, i.p.) strongly reduced the serum glucose levels 1 and 3 h after intraperitoneal injection in alloxan-induced diabetic mice. However, **14** and **16** (l00 mg/kg) lacked hypoglycemic effects. These results indicate that **14** and **16** have neither insulin-like activity nor insulin-releasing activity like tolbutamide, and we speculated that they impact on glucose absorption in the gastrointestinal tract [22].

### Effects on GE in rats

Effects on GE of rats were examined using the phenol red method. The reference drug, atropine sulfate (10 mg/kg, p.o.), can significantly inhibit GE in rats 0.5, l, and 2 h after oral administration. Momordin Ic (**14**) and oleanolic acid 3-*O*-glucuronide (**16**) (50 mg/kg, p.o.) strongly inhibited GE [22]. The GE suppression mediated by **14** and **16** appears critical to suppress the increased serum glucose levels after oral glucose loading.

### Glucose uptake in rat small intestinal fragments in vitro

Next, effects on d-[U-^14^C]glucose (2 mM, 1.0–1.5 × 10^5^ cpm/mL) uptake into small fragments (0.1–0.15 g) of everted rat jejunum at 30℃ for 6 min were examined. Phlorizin (1–100 µM), as a reference compound, inhibited glucose uptake in the rat small intestine in a concentration-dependent manner. Momordin Ic (**14**, 5–500 µM) and oleanolic acid 3-*O*-glucuronide (**16**, 50 and 500 µM) also inhibited uptake in a concentration-dependent manner.

Phlorizin is well-known as an inhibitor of the Na^+^/glucose co-transport system at the intestinal brush border membrane. Both saponins inhibited glucose uptake in rat small intestine fragments in vitro, similar to phlorizin. Based on the above evidence, saponins, such as **14** and **16**, could inhibit the elevated serum glucose levels in oral glucose-loaded rats by suppressing glucose transfer from the stomach to the small intestine, the main site of glucose absorption, and partly by inhibiting glucose transport at the intestinal brush border membrane [22].

Regarding the mode of action of escins Ia (**55**) and Ila (**57**) and *E*,*Z*-senegins II (mixture of **35** and **36**) in mediating inhibitory effects on elevated serum glucose levels in oral glucose-loaded rats, the results were similar to those of oleanolic acid 3-*O*-glycosides (**14** and **16**) [23].

There is insufficient evidence to support that active saponins potently inhibit intestinal absorption in vivo, although they do decrease the increased blood glucose levels in rats by delaying glucose absorption, primarily by inhibiting GE and partly by suppressing the intestinal glucose transport system. It has been reported that reducing postprandial hyperglycemia is an effective strategy for preventing and treating non-insulin-dependent diabetes mellitus. Therefore, these active saponins could also effectively prevent and treat diabetes.

Next, we explored the detailed action of oleanolic acid glycosides and escins Ia–IIb (**55**–**58**) on GE using mice.

## Effects on GE in mice

We examined the effects of oleanolic acid glycosides on GE in non-nutrient meal- or nutrient-meal-loaded mice. Test samples were administered orally to fasted mice at 0.5 h before loading of test meals. Oleanolic acid 3-*O*-monodesmosides [oleanolic acid 3-*O*-glucuronide (**16**, 12.5–50 mg/kg), momordins Ic (**14**, 25–50 mg/kg) and I (**17**, 12.5–50 mg/kg), and 28-*O*-deglucosyl-chikusetsusaponins IV (**19**, 12.5–50 mg/kg) and V (**21**, 50 mg/kg)] exerted inhibitory effects on GE in 1.5% carboxymethyl cellulose sodium salt (CMC-Na) test meal-loaded mice (GE in the control group was ca. 90% and inhibitions were 13.0–57.4, 23.4–63.2, 28.6–87.1, 16.0–65.3, and 20.3%, respectively).

Momordins Ic (**14**) and I (**17**) and 28-*O*-deglucosyl-chikusetsusaponin IV (**19**) (50 mg/kg, p.o.) could also inhibit GE in mice administered 40% glucose test meal (GE in the control group was ca. 65% and inhibitions were 34.1, 45.7, and 26.3%, respectively), milk test meal (GE in the control group was ca. 70% and inhibitions were 39.9, 43.5, and 40.5%, respectively), and 60% ethanol test meal (GE in the control group was ca. 55% and inhibitions were 37.4, 38.5, and 37.6%, respectively). Furthermore, oleanolic acid 3-*O*-glucuronide (**16**) suppressed GE in mice administered the milk test meal and 60% ethanol test meal (57.5 and 37.2%, respectively) but failed to significantly inhibit GE in 40% glucose test meal-loaded mice (10.5%). 28-*O*-Deglucosyl-chikusetsusaponin V (**21**, 50 mg/kg) also slightly inhibited GE in milk test meal-loaded mice (12.5%), but it lacked significant inhibition in mice given 40% glucose or 60% ethanol test meal. Conversely, oleanolic acid 3,28-*O*-bisdesmosides [momordin IIc (**15**), chikusetsusaponins IV (**18**) and V (**20**)], an oleanolic acid 28-*O*-monodesmoside (compound O), and their common sapogenol (oleanolic acid) failed to demonstrate the GE inhibitory effects at 50 mg/kg, and 28-*O*-deglucosyl-chikusetsusaponin V (**21**) showed less inhibition in these experiments [24]. Similarly, glycoside B (**23**, 100 mg/kg) posseing the 2′-*O*-β-d-glucopyranosyl group exhibited weaker activity than glycoside D (**25**) (26.8, 48.5% at 0.5 h) [16].

Capsaicin is widely used to ablate sensory C fibers. It has been systematically used to ablate all capsaicin-sensitive C fiber. Hyperglycemia in streptozotocin-induced hypoinsulinemic rats can reduce the sensitivity of the sympathetic nervous system [25, 26]. In our study, the inhibitory effect against GE in 1.5% CMC-Na test meal-loaded mice was potentiated by glucose [2 g/kg, intravenously (i.v.) or 5 g/kg, i.p.] but markedly attenuated by pretreatment with alloxan (50 mg/kg, i.v.) and streptozotocin (100 mg/kg, i.v.), in which the activity of sympathetic nervous system might be decreased, or by insulin [1 or 3 U/kg, subcutaneously (s.c.)]. The effect of insulin (1 U/kg) was markedly reduced by glucose (2 g/kg, i.v.), which can be directly utilized by the brain, but not by fructose (2 g/kg, i.v.), which cannot be used by the brain [27]. GE is also enhanced by signals from chemoreceptors in severe hypoglycemia, allowing the rapid passage of nutrients through the stomach for immediate digestion and absorption [28]. Pretreatment with capsaicin (75 mg/kg in total, s.c.) could attenuate the effect of momordin Ic (**14**). These results suggest that GE inhibition mediated by **14** is relative to serum glucose levels and partially mediated by capsaicin-sensitive sensory nerves and the central nervous system [27].

Escins Ia–Ilb (**55**–**58**) (12.5–200 mg/kg) inhibited GE of a 1.5% CMC-Na test meal (11.1–52.8%). Treatment with **55**–**58** (50 mg/kg) also inhibited GE of a 40% glucose test meal (21.1–23.5%) except for escin Ia (**55**), a milk test meal (18.4–33.1%), and a 30% ethanol test meal (GE in the control group was *ca*. 70% and inhibitions were 13.5–15.9%). Pretreatment with streptozotocin (100 mg/kg, i.v.), capsaicin (75 mg/kg in total, s.c.), or insulin (1 U /kg, s.c.) could attenuate the effects of **55**–**58** on GE of the CMC-Na test meal. The effect of insulin was reduced by glucose (2 g/kg, i.v.), which can be directly used by the brain, but not by fructose (2 g/kg, i.v.), which cannot be utilized by the brain. The inhibitory effects of **55**–**58** (50 mg/kg) could not be observed on the GE of 60% ethanol test meal, in which the central nervous system was suppressed by ethanol. Accordingly, capsaicin-sensitive sensory nerves and the central nervous system may partially mediate the effects of **55**–**58** [29]. Furthermore, the GE inhibitory effects mediated by **55**–**58** (25 mg/kg) were markedly attenuated following pretreatment with indomethacin, an inhibitor of prostaglandins (PGs) biosynthesis, suggesting the involvement of endogenous PGs in GE inhibition [30].

Dopamine (DA) is a major neurotransmitter in the central nervous system. DA is also found in large concentrations in the stomach and is suggested to be involved in controlling GE in rats. Escin Ib (**56**, 25 mg/kg, p.o.) mediated GE inhibition was attenuated following pretreatment with a single bolus of dl-α-methyl-*p*-tyrosine methyl ester (an inhibitor of tyrosine hydroxylase), reserpine (a catecholamine depletor), 6-hydroxydopamine (a dopamine depletor). Furthermore, pretreatment with centrally-acting DA_2_ receptor antagonists (e.g., spiperone, haloperidol, metoclopramide) attenuated the effect of **56**. However, a peripherally-acting DA_2_ antagonist, domperidone, exerted weak attenuation, whereas SCH23390 (a DA_1_ receptor antagonist) did not [31]. These findings suggest that **56** could inhibit GE, at least in part, mediated via capsaicin-sensitive sensory nerves, to stimulate the synthesis and/or release of DA, to act through the central DA_2_ receptor, which, in turn, causes PGs synthesis or release.

## Effects on gastrointestinal transit in mice

Ileus is a common complication induced by various factors, such as laparotomy with manipulation and peritoneal irritation. Given the lack of specific therapy, ileus remains an important clinical challenge. Patients with ileus accumulate gas and secretions, leading to bloating, distention, emesis, and pain. Prokinetic drugs, such as cisapride, metoclopramide, erythromycin, and octreotide, are commonly used to combat chronic ileus. However, no medical therapy affords notable relief in advanced cases. Non-steroidal anti-inflammatory drugs, such as indomethacin, are known to block PG biosynthesis and are widely used for postoperative pain. These drugs have been shown to afford beneficial effects in treating postoperative ileus in rodents, although undesirable side effects have also been documented. Recently, a Kampo preparation, daikenchuto (大建中湯), has been used clinically to treat ileus post-abdominal surgery, and the effective constituents and detailed mechanisms of action have been revealed [32].

Screening can be performed using a 5% charcoal suspension in a 1.5% CMC-Na solution intragastrically administered (0.2 mL/mouse) to conscious mice. Thirty minutes later, mice were sacrificed by cervical dislocation. The abdominal cavity was opened, and the gastrointestinal tract was harvested. The distance traveled by the front of charcoal suspension from the pylorus was measured and expressed as a percentage of the total length of the small intestine from the pylorus to the caecum. In this condition, GIT in the control group was ca. 50%. The test samples were administered orally 60 min prior to administering the charcoal suspension.

First, the effects of oleanolic acid glycosides on the GIT of ileus were examined in normal fasted mice. One hour after oral administration, three oleanolic acid 3-*O*-monodesmosides [oleanolic acid 3-*O*-glucuronide (**16**), momordins Ic (**14**) and I (**17**)] (50 mg/kg) significantly accelerated GIT with acceleration rates of 42.6, 44.4, and 37.3%, while two oleanolic acid 3-*O*-monodesmosides [28-*O*-deglucosyl-chikusetsusaponins IV (**19**) and V (**21**)], oleanolic acid 3,28-*O*-bisdesmosides [momordin IIc (**15**), chikusetsusaponins IV (**18**) and V (**20**)], and their common sapogenol (oleanolic acid) (50 mg/kg, p.o.) failed to yield any notable effect. Conversely, an oleanolic acid 28-*O*-monodesmoside (compound O) (50 mg/kg, p.o.) inhibited GIT by 24.8% [33].

Ileus was induced by peritoneal irritation or by laparotomy with manipulation. In our experiments, GIT could be suppressed by peritoneal injection of 1% acetic acid and laparotomy with manipulation; the GITs in the control groups were ca. 14 and 23%, respectively. Momordins Ic (**14**, 5–25 mg/kg) and I (**17**, 25 mg/kg) also significantly accelerated the reduced GIT induced by the intraperitoneal acetic acid injection, with acceleration rates of 109.2–246.8 and 63.4%, respectively. In contrast, compound O and chikusetsusaponin V (**20**) (50 mg/kg) potentiated the suppressed GIT by 36.4 and 40.3%, whereas oleanolic acid, oleanolic acid 3-*O*-glucuronide (**16**), momordin IIc (**15**), chikusetsusaponins IV (**18**) and V (**20**), and 28-*O*-deglucosyl-chikusetsusaponins IV (**19**) and V (**21**) (50 mg/kg) showed no significant effect. Oleanolic acid 3-*O*-glucuronide (**16**), momordins Ic (**14**) and I (**17**), and 28-*O*-deglucosyl-chikusetsusaponins V (**21**) (50 mg/kg) significantly accelerated the reduced GIT induced by laparotomy with manipulation with acceleration rates of 52.3–63.7%, while oleanolic acid, compound O, momordin IIc (**15**), chikusetsusaponins IV (**18**) and V (**20**), and 28-*O*-deglucosyl-chikusetsusaponins IV (**19**) (50 mg/kg) showed no significant effect [33].

The oleanolic acid 3-*O*-glycosides (**14**, **16**, and **17**)-mediated accelerated GIT was completely abolished by the pretreatment with streptozotocin (100 mg/kg, i.v.) but not by the pretreatment with capsaicin (75 mg/kg in total, s.c.). These results suggest that the sympathetic nervous system, not capsaicin-sensitive sensory nerves, may mediate the enhanced GIT induced by oleanolic acid 3-*O*-glycosides (**14**, **16**, and **17**) [33].

The effects of escins Ia–IIb (**55**–**58**) on GIT and ileus, as described above, were investigated in mice. Compounds **55**–**58** (25–50 mg/kg) dose-dependently accelerated GIT in normal mice (acceleration rate: 19.6–38.8%). Compounds **55**–**58** (25–50 mg/kg) dose-dependently accelerated the reduced GIT induced by intraperitoneal acetic acid irritation (acceleration rate: 69.0–213.0%) and the reduced GIT mediated by laparotomy with manipulation (acceleration rate: 37.3–89.0%). Desacylescins I and II (50 mg/kg) showed no such effects [34].

In this experiment using normal mice, the applied interval between the saponin fraction and charcoal suspension was set from 5 to 300 min. Interestingly, the saponin fraction (25 mg/kg) demonstrated significant effects 5 min after the oral administration, which persisted until 240 min. These findings suggest that the saponin act immediately after oral administration, with actions persisting for 4 h [34].

The GIT acceleration induced by **55**–**58** in normal mice was completely abolished by the pretreatment with streptozotocin but not by the pretreatment with capsaicin (75 mg/kg in total, s.c.) or atropine (10 mg/kg, s.c.). Accordingly, these results suggest that the sympathetic nervous system, but not capsaicin-sensitive sensory nerves nor the cholinergic mechanism, mediates the GIT accelerations induced by **55**–**58**, similar to oleanolic acid 3-*O*-glycosides (**14**, **16**, and **17**). The GIT acceleration induced by **55**–**58** may be mediated by the release of endogenous PGs and nitric oxide (NO), as determined by the results of pretreatment with indomethacin and NO synthase (NOS) inhibitor [*N*^G^-nitro-l-arginine methyl ester (l-NAME)] [30]. Furthermore, the GIT acceleration mediated by escin Ib (**56**, 25 or 50 mg/kg, p.o.) was attenuated following pretreatment with 5-HT_2_ receptor antagonists (e.g., ritanserin, ketanserin, haloperidol, spiperone), but not by 5-HT_3_ or 5-HT_4_ receptor antagonists (MDL72222, metoclopramide or tropisetron). A bolus of *dl*-*p*-chlorophenylalanine methyl ester (an inhibitor of 5-HT synthesizing enzyme, tryptophan hydroxylase) and reserpine (a 5-HT depletor), but not 6-hydroxydopamine (a dopamine depletor), could attenuate the GIT acceleration effects of **56** [35]. In addition, we reported that chakasaponin II (**78**), classified into the acylated polyhydroxyolean-12-ene 3-*O*-monodesmoside like escins, stimulated the release of 5-HT from intestinal fragments in vitro, as described in the section of tea-flower.

Collectively, these saponins stimulate the synthesis or release of 5-HT to act through 5-HT_2_ receptors, possibly the 5-HT_2A_ receptor, which, in turn, causes the release of NO and PGs, thereby accelerating GIT at the intestinal tract. The potential therapeutic effects of saponins should be explored in clinical settings for preventing the inhibition of GIT, including ileus induced by peritoneal irritation.

## Gastromucosal protective effects in rats

As a beneficial effect against alcohol toxicity, we examined the effects of oleanolic acid glycosides on ethanol-induced gastric mucosal lesions in rats. In addition, the effects of oleanolic acid glycosides on indomethacin-induced gastric lesions and gastric secretion in pylorus-ligated rats were examined. The lesions were characterized by multiple hemorrhage red bands (patches) of different sizes along the long axis of the glandular stomach. The total length (mm) or score of lesions of each rat was measured 1 h after the administration of 99.5% ethanol (1.5 mL/rat, p.o.) or 4 h after the administration of indomethacin (30 mg/kg, s.c.). Test samples were administered orally to fasted rats 1 h prior to treatment with ethanol, indomethacin, or pyloric ligation.

Oleanolic acid 3-*O*-monodesmosides [momordin Ic (**14**, 10–50 mg/kg), oleanolic acid 3-*O*-glucuronide (**16**, 20–50 mg/kg), and 28-*O*-deglucosyl-chikusetsusaponins IV (**19**, 10–50 mg/kg) and V (**21**, 10–50 mg/kg), and glycosides B (**23**, 20 mg/kg) and D (**25**, 20 mg/kg)] afforded protective effects against ethanol-induced gastric mucosal lesions (inhibition: 76.7–99.7, 71.3–96.4, 62.6–94.3, 80.4–100.0, 80.8, and 82.4%, respectively), whereas oleanolic acid 3,28-*O*-bisdesmosides [momordin IIc (**15**), chikusetsusaponins IV (**18**) and V (**20**)] except for glycoside C (**24**, 20 mg/kg) (inhibition: 44.9%), an oleanolic acid 28-*O*-monodesmoside (compound O), and their common sapogenol (oleanolic acid) failed to induce such effects. Moreover, oleanolic acid 3-*O*-monodesmosides [**14** (2.5–50 mg/kg), **16** (20–50 mg/kg), and **19** (20–50 mg/kg)] exerted protective effects against indomethacin-induced gastric mucosal lesions (inhibition: 46.2–97.4, 47.4–83.5, and 48.6–85.5%, respectively). However, 28-*O*-deglucosyl-chikusetsusaponin V (**21**) and glycoside B (**23**) afforded no gastroprotection, whereas chikusetsusaponin V (**20**, 20–50 mg/kg) and glycoside A (**22**, 20 mg/kg) induced gastroprotective effect (inhibition: 67.9–80.5 and 75.2%). Based on the findings of the indomethacin-induced injury, the mechanism of action of **20** and **22** possessing the 2′-*O*-β-d-glucopyranosyl group might differ from that of the other active saponins [16, 36].

Escins Ia–IIb (**55**–**58**) also exerted a potent protective effect against ethanol-induced gastric lesions in rats, dose-dependently reducing the lesion scores at doses of 5–50 mg/kg (inhibition: 41.8–99.0%) and improving the pathogenic changes. Conversely, desacylescins I and II (50 mg/kg) had no such effect [37].

Pyloric ligation for 3 h resulted in gastric acid accumulation*.* Momordin Ic (**14**), oleanolic acid 3-*O*-glucuronide (**16**), 28-*O*-deglucosyl-chikusetsusaponins IV (**19**) and V (**21**), and chikusetsusaponin V (**20**) did not decrease gastric secretion at examined doses. In contrast, **14** and **16** (20–50 mg/kg) significantly increased gastric secretion (volume, acid and pepsin outputs). These findings indicated that the protective activities of these active saponins are acid-independent [36]. Notably, escins Ia–IIb (**55**–**58**) (10 and 20 mg/kg) did not decrease the gastric secretion but tended to increase the gastric secretion without altering the pH value of gastric juice [37].

PGs and NO as well as capsaicin-sensitive neurons have been shown to participate in the gastric defense mechanism. Oxygen-derived free radicals and lipid peroxidation are associated with gastrointestinal lesions, and antioxidants prevent the lesions by various ulcerogens. Gastric mucosal sulfhydryls (SHs) including glutathione (GSH) act as antioxidants, and are important for maintenance of mucosal integrity in the stomach. Ethanol-induced gastric damage is also associated with a significant decrease in the mucosal SHs level such as GSH, and pretreatment with SH-blockers prevents the gastroprotection of SH-containing compounds [38].

The gastroprotective effects of **16** and **55**–**58** were attenuated following pretreatment with capsaicin, L-NAME (70 mg/kg, i.p.), and indomethacin (10 mg/kg, s.c.), but not by *N*-ethylmaleimide (10 mg/kg, s.c.), a SHs blocker. The effects of **55**–**58** were also attenuated in streptozotocin-induced diabetic rats. Based on these findings, it can be suggested that the gastroprotective effects of **55**–**58** on ethanol-induced gastric mucosal lesions are acid-independent, potentially mediated by endogenous PGs, NO, and capsaicin-sensitive sensory nerves. Furthermore, the sympathetic nervous system partly mediates these effects, although the underlying mechanism remains unclear [36, 37, 39].

## Antipruritic and anti-inflammatory effects

The fruit of *K. scoparia* has been used to treat skin diseases and cutaneous pruritus in Chinese traditional medicine. Matsuda et al. reported that the 70% aqueous ethanol extract of this natural medicine and its principal saponin constituent, momordin Ic (**14**), exerts antiallergic, anti-inflammatory, and antinociceptive effects. Furthermore, the 70% aqueous ethanol extract and **14** exhibited antipruritic activity, as determined by its inhibitory effect on the compound 48/80-induced pruritic mouse model [40]. As a continuing study, we examined the antipruritic activity of oleanolic acid glycosides.

Oleanolic acid 3-*O*-monodesmosides [oleanolic acid 3-*O*-glucuronide (**16**, 0.2 mmol/kg), momordin Ic (**14**, 0.2 mmol/kg) and its 2′-*O*-β-d-glucopyranoside (0.1 mmol/kg), and momordin I (**17**) (0.13 mmol/kg)] could suppress the scratching induced by compound 48/80 in mice (inhibition: 52.4, 51.8, 43.7, and 58.9%); however, oleanolic acid 3,28-*O*-bisdesmoside [momordin IIc (**15**, 0.11 mmol/kg), chikusetsusaponin V (**20**, 0.2 mmol/kg)] lacked this activity. Furthermore, among oleanolic acid 3-*O*-monodesmosides, oleanolic acid 3-*O*-β-d-glucopyranoside showed less activity than **16** [41].

The saponin mixture ‘escin’ obtained from the seeds of the horse chestnut seeds could afford anti-inflammatory activity [42]; however, the anti-inflammatory effects of each pure saponin of escin had not been examined, given the incomplete isolation and structural determination of saponin constituents.

We explored the effects of escins Ia–Ilb (**55**–**58**) and desacylescins I and II on acute inflammation in animals. All escins (50–200 mg/kg, p.o.) suppressed the increased vascular permeability induced by both intraperitoneal injection of 1% acetic acid in mice (inhibition: 12.1–56.5%) and intracutaneous (i.c.) injection of histamine (100 µg/site) in rats (25.0–74.3%). In addition, escins Ib–IIb (**56**–**58**) (50–200 mg/kg) inhibited serotonin (2.5 µg/site, s.c.)-induced vascular permeability in rats (36.1–86.2%), but **55** did not show significant inhibition. All escins (200 mg/kg) inhibited carrageenin-induced hind paw edema during the first phase in rats. Escin Ia (**55**) (200 mg/kg) and **56**–**58** (50–200 mg/kg) inhibited the scratching behavior induced by compound 48/80 in mice (inhibition: 39.2–80.5%), although **55** afforded the weakest inhibition (42.4% at 200 mg/kg). Desacylescins I and II (200 mg/kg) did not afford inflammatory effects [43].

Our hypothesis of the potential mechanism of action saponins was established based on the experimental results using various receptor inhibitors and activators, inhibitors of NO and PG biosynthesis, and a high dose of capsaicin to mainly damage the afferent vagal nerves; however, these agents act systemically and not selectively act at each tissue and nerve. Therefore, further experiments, such as determining the concentrations of catecholamines in the brain and intestine using microdialysis-HPLC methods and selective detection of afferent vagal nerves, need to be considered. The interpretation of our experimental results should be reconsidered, given that regulation of gastrointestinal movements has been updated in detail [28, 38, 44, 45].

## Structure–activity relationship

Here, we summarize the structural requirements of active saponins to suppress elevated blood alcohol and glucose levels in rats, inhibit GE and accelerate GIT in mice, and afford gastromucosal protection in rats, as well as antipruritic and anti-inflammatory activities; however, detailed structure–activity relationships of saponins remain poorly clarified based on our reported results.

### Inhibition of elevated blood alcohol and glucose levels

Regarding the inhibition of elevated blood alcohol levels, the 3-*O*-glycoside moiety and 28-carboxyl group in oleanolic acid glycosides are essential for mediating this activity [5, 10]. The 28-*O*-glucopyranosyl group or the 2′-*O*-β-d-glucopyranosyl group can reduce the suppression of elevated blood alcohol levels.

Acyl groups, such as 4-methoxycinnamoyl group at the 28-*O*-oligoglycoside moiety of senegasaponins and senegins, the 22-*O*-tigloyl or angeloyl group of camelliasaponins, the 21-*O*-tigloyl or angeloyl and 22-*O*-acetyl groups of escins, are essential for the inhibition, as their desacyl derivatives lacked activity [11–14].

Comparing the 28-oligoglycoside structures and the inhibitory activities of *E*,*Z*-senegin II (**35**, **36**) and *E*,*Z*-senegasaponins a (**41**, **42**) and b (**43**, **44**) with those for inactive *E*,*Z*-senegins III (**37**, **38**) and IV (**39**, **40**), we speculated that the α-l-rhamnopyranosyl group linked to the fucopyranosyl moiety in senegins could reduce the inhibitory activity. The 2′-*O*-β-d-xylopyranosyl group at the 3-*O*-oligoglycoside moiety of escins is required for potent inhibitory activity, and the 2′-*O*-β-d-glucopyranosyl group could reduce the inhibitory activity [11, 12, 14].

Regarding the inhibitory effects of oleanolic acid glycosides on elevated blood glucose levels, oleanolic acid 3-*O*-monodesmosides and 3,28-*O*-bisdesmosides, such as **13** and **18**, comprised of a 4′-*O*-l-arabinofuranosyl group, which tends to inhibit elevated plasma glucose levels; this action appears distinct from inhibitory effects mediated by oleanolic acid glycosides on elevated ethanol levels mediated [5, 6, 10]. The 2′-*O*-β-d-glucopyranosyl group of an oleanolic acid 3-*O*-monodesmoside [glycosides B (**23**)] also could reduce the inhibitory activity similar to the inhibition of elevated blood ethanol levels [16].

Similar to the inhibition of elevated blood ethanol levels, acyl groups of senegasaponins, senegins, and escins are essential for inhibiting elevated blood glucose levels, and the 2′-*O*-β-d-xylopyranosyl group of escins is responsible for mediating potent inhibitory activity; the 2′-*O*-β-d-glucopyranosyl group reduces this inhibitory activity [11, 12, 14].

### GE inhibition and GIT acceleration

The 3-*O*-monodesmoside structure and 28-carboxyl group are essential for GE inhibition in mice, and the 28-ester glucoside moiety and 2′-*O*-β-d-glucopyranosyl group reduce this inhibitory activity [24], similar to the inhibitory effects on elevated blood ethanol and glucose levels in rats. All escins Ia–IIb (**55**–**58**) could inhibit GE of a 1.5% CMC-Na test meal, a 40% glucose test meal, a milk test meal, and a 30% ethanol test meal-loaded mice, except for the effect of escin Ia (**55**) possessing the 2′-*O*-β-d-glucopyanosyl group on GE of 40% glucose test meal-loaded mice. The presence of the 2′-*O*-β-d-glucopyranosyl group did not markedly reduce activity [29].

As observed for GE inhibitory effects, the 3-*O*-monodesmoside structure and 28-carboxyl group of oleanolic acid glycosides are essential for accelerating GIT in mice, except for 28-*O*-deglucosyl-chikusetsusaponins IV (**19**) and V (**21**) [33]. Escins could accelerate the reduced GIT mediated by intraperitoneal acetic acid administration and laparotomy with manipulation, and the 21,22-acyl groups are essential for activity [34].

### Gastromucosal protection

The 3-*O*-glycoside moiety of oleanolic acid glycosides was found to be essential for suppressing ethanol-induced gastric lesions, and the 28-ester glucoside could reduce this inhibitory activity. Furthermore, the 2′-*O*-β-d-glucopyranosyl group of the glucuronic acid part decreased the activity, similar to the effects on elevated blood alcohol and glucose levels [36]. The 21,22-acyl groups of escins are crucial for affording protection against ethanol-induced gastric lesions, similar to their function in mediating the other observed effects, while the 2′-*O*-β-d-glucopyranosyl group did not markedly reduce activity, as described in the section of GE and GIT [37].

### Antipruritic and anti-inflammatory effects

Regarding the relationship between their chemical structures and activities, the 3-*O*-glycoside moiety and the 28-carboxyl group of oleanolic acid glycoside were found to be essential for exerting the antipruritic effects, similar to the effects described in other sections, and the 3-*O*-glucuronide showed more potent activity than the corresponding 3-*O*-glucoside [41].

The acyl groups of escins are essential for exerting anti-inflammatory effects, as described in other sections. Furthermore, escins Ib–IIb (**56**–**58**) with either the 21-*O*-angeloyl group or the 2′-*O*-d-xylopyranosyl group showed more potent activities than **55** with both the 21-*O*-tigloyl and the 2′-*O*-β-d-glucopyranosyl groups [43].

**Fig. 4:**
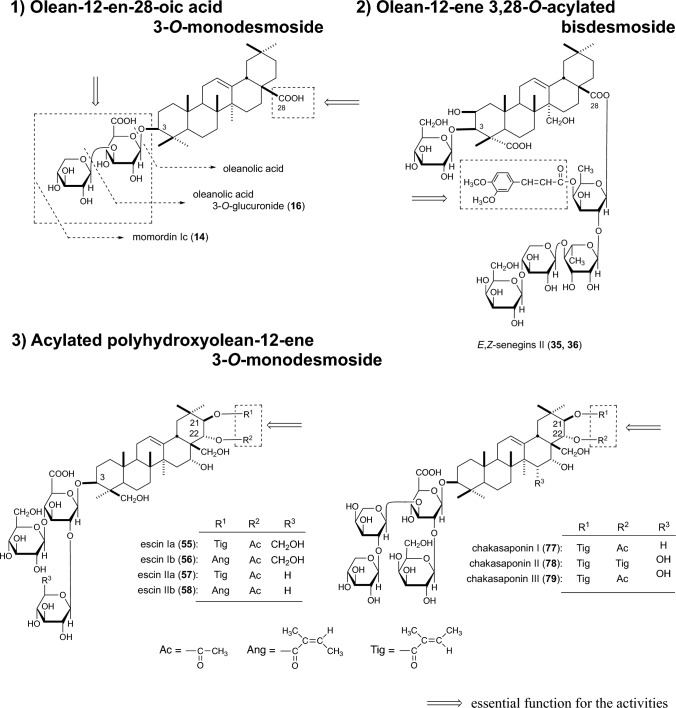
Three type of the active oleanane-type triterpene saponins

As described earlier, structural requirements of oleanolic acid glycosides and escins are similar for affording GE inhibition, GIT acceleration, gastroprotection, and anti-inflammation, although several exceptions were observed. Based on the common structural requirements, the active oleanane-type triterpene saponins could be classified into the following there types: 1) olean-12-en-28-oic acid 3-*O*-monodesmoside, 2) olean-12-ene 3,28-*O*-acylated bisdesmoside, and 3) acylated polyhydroxyolean-12-ene 3-*O*-monodesmoside (Fig. 4).

Furthermore, common modes of action, such as involvements of capsaicin-sensitive nerves, endogenous NO and PGs, and possibly sympathetic nerves, as well as common structural requirements, were observed. Based on these findings, a common mechanism of action might mediate the pharmacological effects of active saponins.

## Anti-obesity effects of tea-flower and appetite inhibition in mice

Tea prepared from leaves of the plant *C. sinensis* (tea leaves) has been used since ancient days for medicinal purposes and is now consumed as a popular beverage. Tea has been extensively explored for its beneficial health effects, such as reducing body weight, alleviating metabolic syndrome, preventing cardiovascular diseases and cancer, and protecting against neurodegeneration. Regarding the mechanisms responsible for benefits against metabolic syndrome, tea polyphenols such as (-)-epigallocatechin 3-*O*-gallate can reduce intestinal lipid absorption, as well as activate AMP-activated protein kinase in the liver, skeletal muscle, and adipose tissues. The activation of AMP-activated protein kinase decreases gluconeogenesis and fatty acid synthesis and increases catabolism, resulting in body weight reduction and alleviation of metabolic syndrome [46–48].

Although the biofunctional effects of tea leaves have been extensively investigated, flowers and seeds of the tea plant remain poorly explored. We have reported various tea saponins and assamsaponins, classified as acylated oleanane-type triterpene oligogycosides, from the seeds of *C. sinensis* and *C. sinensis* var. *assamica* [49–56]. Among them, theasaponins E_1_, E_2_, E_5_, and assamsaponin C (at a low dose of 5.0 mg/kg) exerted protective effects against ethanol-induced gastric mucosal lesions in rats [50, 52, 53], and theasaponin E_1_ inhibited GE and accelerated GIT [51], similar to escins. Foliatheasaponins, derived from the leaves of Japanese *C. sinensis* (Tencha), exerted an antiallergic effect in vitro [57].

**Fig. 5:**
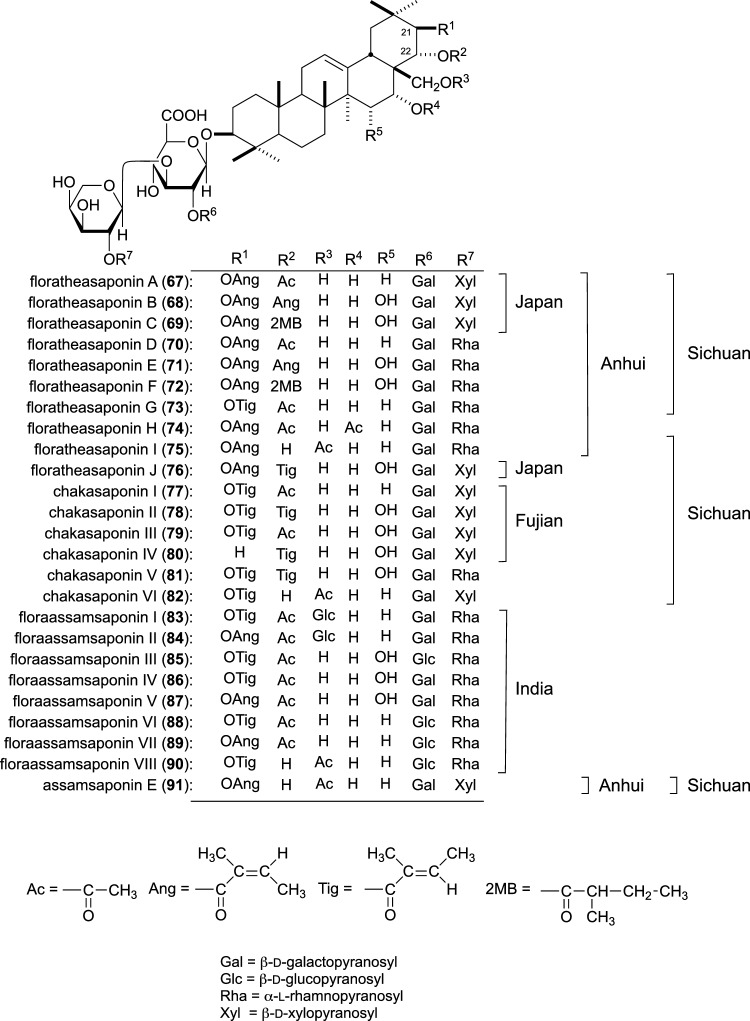
Chemical structures of floratheasaponins A–J (**67**–**76**), chakasaponins I–VI (**77**–**82**), floraassamsaponins I–VIII (**83**–**90**), and assamsaponin E (**91**) from tea-flower. This figure was taken from reference [85] with a modification

Regarding the biofunctional effects of tea-flower (‘Chaka’ in Japanese), we reported the antihyperlipidemic [58, 59], antihyperglycemic [59], GE inhibitory [59], gastroprotective [60], anti-obesity effects [61], and GIT accelerative [62] in vivo, along with antiallergic [63] and inhibitory effects on pancreatic lipase [62] and amyloid β (Aβ) aggregation [64] of the extract. Overall, we identified 24 new saponins, floratheasaponins A–J (**67**–**76**), chakasaponins I–VI (**77**–**82**) together with assamsaponin E (**91**) from tea-flower collected in Anhui, Sichua, and Fujian provinces (Anhui, Sichuan, and Fujian Chaka) and Japan (Japanese Chaka) [58–63, 65], and floraassamsaponins I–VIII (**83**–**90**) from the flower buds of *C. sinensis* var. *assamica* in India (Indian Assam Chaka)[64] (Fig. 5). Furthermore, we isolated several flavonol glycosides from each tea-flower [63, 66], and quantitatively analyzed saponins and flavonol glycosides [67–69].

Herein, we focus on the anti-obesity effects of tea-flower collected in the Fujian province of China (Fujian Chaka) and saponin constituents with anti-appetite effects.

**Fig. 6:**
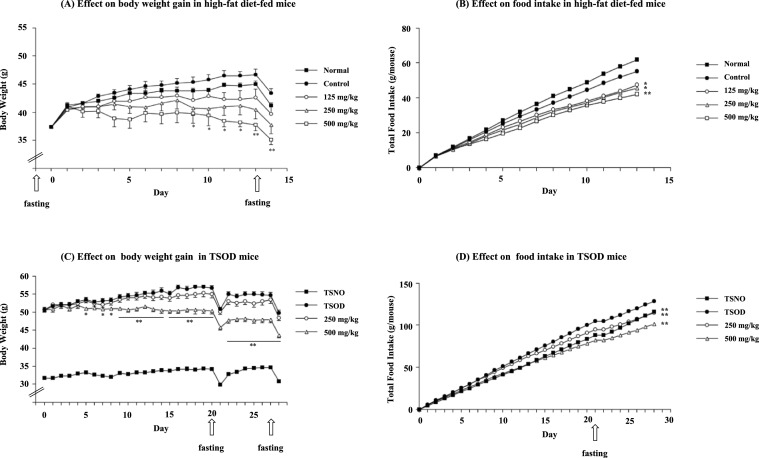
Effects of the methanolic (MeOH) extract of Fujian Chaka on body weight gain and food intake in high-fat diet-fed mice and TSOD mice. **A** Effects on body weight (g) of ddY mice fed a high-fat diet (45 kcal% fat, D12451; Research Diet, Inc.) or normal diet (10 kcal% fat, D12450B; Research Diet, Inc.) for 14 days. **B** Effects on food intake of the high-fat diet in ddY mice. **C** Effects on body weight (g) of TSOD mice fed a standard laboratory chow (MF, Oriental Yeast Co., Ltd.). **D** Effects on food intake of the standard laboratory chow in TSOD mice.The test sample was administered orally once daily. Each value represents the mean with the standard error of the mean (S.E.M.) (*n* = 6–10, *p < 0.05 **p < 0.01). This graph was taken from reference [61] with a modification

The effects of the methanol (MeOH) extract on body weight gain in high-fat diet-fed mice and an experimental animal of metabolic syndrome, TSOD (Tsumura Suzuki Obese Diabetic) mice, were examined [61]. The MeOH extract (500 mg/kg/day, p.o*.*) markedly inhibited body weight gain 9–14 days after administration to high-fat diet-fed mice (Fig. 6A). After two weeks, treatment with the extract (500 mg/kg/day, p.o*.*) significantly suppressed liver weight (*p* < 0.05, 1.06 g vs. control 1.23 g), liver triglycerides (*p* < 0.01, 30.2 mg/g wet tissue *vs*. control 62.1 mg/g wet tissue) and the weight of visceral fat (*p* < 0.05, 1.70 g vs. control 2.73 g). After one week of administration, the extract (500 mg/kg/day, p.o.) also significantly suppressed body weight gain in TSOD mice (Fig. 6C). Three weeks later, a glucose tolerance test was performed by intraperitoneal injection of glucose. The MeOH extract (250 and 500 mg/kg/day, p.o.) significantly suppressed increased plasma glucose levels 2 h after glucose loading. After four weeks, treatment with the extract (500 mg/kg, p.o*.*/day) significantly suppressed liver weight (*p* < 0.01, 1.27 g vs. control 1.48 g), weight of visceral fat (*p* < 0.01, 3.67 g vs. control 5.23 g), and plasma total cholesterol levels (*p* < 0.05, 210.4 mg/dL *vs*. control 254.3 mg/dL).

We speculated that the potent reduction in body weight within one week of extract treatment could be primarily attributed to reduced food intake. Therefore, the effect of the extract on food intake was examined in high-fat diet-fed and TSOD mice (Figs. 6B, D). The extract inhibited food intake in a dose-dependent manner, and this effect was also observed in normal diet-fed mice; the total intake for 5 days in the MeOH extract-treated group (500 mg/kg/day, p.o.) was 19.3 g (*p* < 0.01) vs. 21.0 g in the control group, although no obvious toxic effect was observed except for body weight gain [61].

The *n*-BuOH-soluble fraction inhibited food intake at a dose of 250 mg/kg/day, p.o., but the EtOAc- and H_2_O-soluble fractions had no such effect when administered orally according to yield.

Regarding the effect of the *n*-BuOH-soluble fraction on appetite signals, the effects on hypothalamic mRNA expression of neuropeptide Y (NPY) and agouti-related protein (AgRP) were examined. NPY is an important regulator of body weight that mediates its effects on food intake and energy expenditure. Several neurons expressing NPY in the hypothalamus are found within the arcuate nucleus (ARC), with most co-expressing AgRP. Ablation of NPY/AgRP neurons in young mice was shown to reduce food intake and body weight, and intracerebroventricular (i.c.v.) injection of NPY potently stimulated food intake in adult rats [70]. In our study, the *n*-BuOH-soluble fraction administrated at 250 mg/kg for 4 days significantly suppressed NPY mRNA expression. These findings suggest that the *n*-BuOH-soluble fraction inhibited food intake by suppressing appetite signals.

Furthermore, a principal saponin, chakasaponin II (**78**) (50 mg/kg/day, p.o.), induced a suppressive effect on food intake and the hypothalamic expression of NPY mRNA levels, similar to the *n*-BuOH-soluble fraction. These results suggest that the saponins are active constituents of the extract. Furthermore, the desacyl derivative of **78**, desacyl-floratheasaponin B, failed to exert these suppressive effects, suggesting that the 21 and 22-acyl groups are critical for the activity, as observed for the other effects described in the section of structure-activity relationship.

**Fig. 7:**
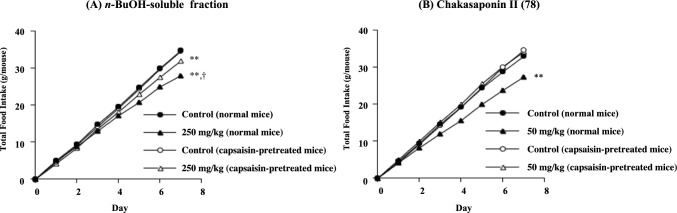
Effects of the *n*-BuOH-soluble fraction and chakasaponin II (**78**) on food intake of standard laboratory chow in normal mice and/or capsaicin-pretreated mice. Male ddY mice were fed a standard laboratory chow (MF, Oriental Yeast Co., Ltd.) for 8 days. The test sample was administered orally once daily. Each value represents the mean for 5 or 6 mice. Significantly different from the control, **p < 0.01, and from the corresponding capsaicin-treated group, ^†^*p* < 0.05. This graph was taken from reference [61] with a modification.

Recently, an anti-cancer drug, cisplatin, and selective serotonin reuptake inhibitors (SSRIs) were found to inhibit food intake, and the involvement of 5-HT_2_ receptors in appetite control has been reported. Activation of the 5-HT_2B_ receptor in gastric smooth muscle and the 5-HT_2C_ receptor in the hypothalamus can suppress appetite. 5-HT produced during cisplatin or SSRI treatment binds to various receptor subtypes and is likely to stimulate the 5-HT_2B_ and 5-HT_2C_ receptors. Stimulating the 5-HT_2B_ receptor decreases plasma ghrelin levels, suppressing the appetite signals via afferent vagal nerves [71–73]. Consistent with previous reports, 5-HT (1 mg/kg, i.p.) inhibited food intake in mice. We investigated 5-HT release from isolated ilea of mice and its tissue retention in vitro. Chakasaponin II (**78**) at 1.0 mM significantly enhanced 5-HT release into the medium and reduced tissue retention [61]. The concentration of **78** was relatively high for in vitro experimentation, but saponin concentrations are typically considered to be elevated in the intestinal tract, given that this type of compound is poorly absorbed [42, 74]. In our preliminary investigation, more than 30% of **78** was retained in the small intestinal tract 1 h after oral administration to mice. Furthermore, the effects of the *n*-BuOH-soluble fraction and chakasaponin II (**78**) on food intake were notably reduced in capsaicin-pretreated mice in which the capsaicin-sensitive sensory nerves were desensitized by pretreatment with high-dose capsaicin (Fig. 7) similar to that observed with escins (**55**–**58**) [61].

**Fig. 8:**
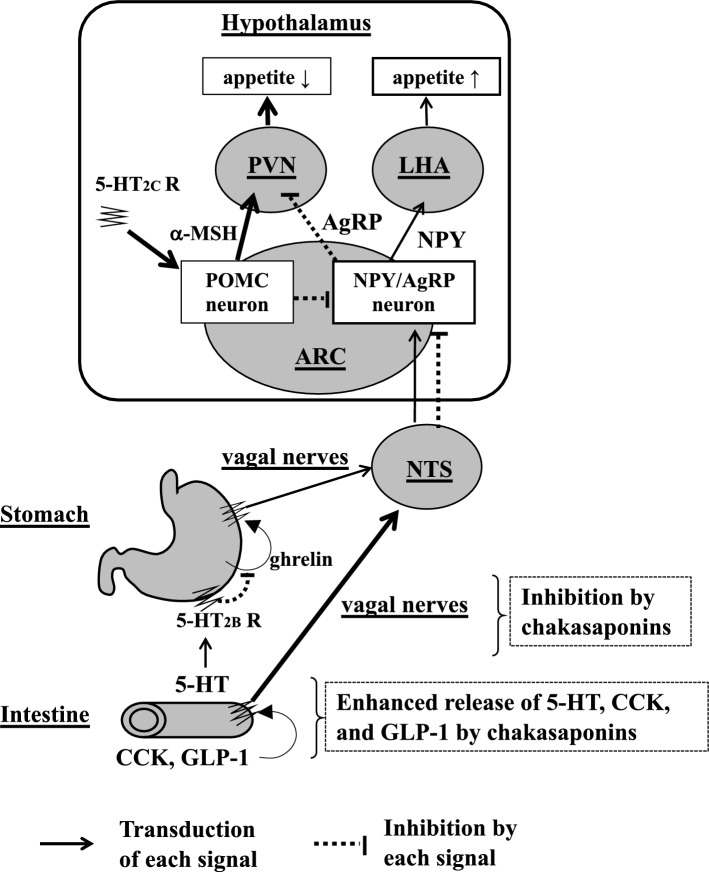
Appetite signals in the gastrointestinal-brain system. *NPY* neuropeptide Y, *AgRP* agouti-related protein, *MSH* melanocyte-stimulating hormone, *POMC* proopiomelanocortin, *NTS* nucleus tractus solitarius, *ARC* arcuate nucleus, *PVN* paraventricular nucleus, *LHA* lateral hypothalamic area, *CCK* cholecystokinin, *GLP-1* glucagon-like peptide 1. This figure is taken from reference [85] with a modification

Cholecystokinin (CCK) and glucagon-like peptide 1 (GLP-1) secreted from the intestinal I-cells and L-cells stimulate each receptor, and the signals are mediated through the afferent vagal nerves and nucleus tractus solitarius (NTS) to reduce the expression of NPY and AgRP, ultimately suppressing appetite. Stimulation of the 5-HT_2B_ receptor in the stomach via the 5-HT released from intestinal chromaffin cells inhibits the release of ghrelin, which stimulates the appetite through the afferent vagal nerves, and stimulation of the 5HT_2C_ receptor in the hypothalamus stimulates proopiomelanocortin (POMC) neurons to reduce appetite [70–75]. In our preliminary examination, chakasaponin II (**78**) increased plasma CCK and GLP-1 levels in mice. These findings suggest that their inhibitory effects on food intake were initiated by the excretion of CCK and GLP-1, and mediated via capsaicin-sensitive sensory nerves, probably the afferent vagal nerves (Fig. 8). Chakasaponins I–III (**77**–**79**) (50–100 mg/kg) inhibited plasma glucose levels after sucrose loading in mice without inhibiting intestinal α-glucosidase and suppressed GE, similar to escins. CCK and GLP-1 were shown to inhibit GE [76, 77], suggesting that CCK and GLP-1 release also participates in GE inhibition meditated by saponins such as chakasaponins and escins [29, 59].

As described in the section of effects on GE in mice, the inhibitory effects of escins on GE involved DA release and DA_2_ receptors via mechanisms involving capsaicin-sensitive sensory nerves, probably certain vagal afferent nerves [29, 31]. Tominaga et al. reported that 5-HT inhibits GE in rats [78]. Consistently, 5-HT (10 mg/kg, i.p.) significantly inhibited GE under our experimental conditions, although the effective dose of 5-HT was higher than that for food intake. Pretreatment with capsaicin partly reduced the inhibitory effects of chakasaponins I (**77**) and II (**78**) on GE, suggesting that certain afferent vagal nerves, at least in part, participate in the inhibition of GE and food intake [61]. Bugajski et al. have observed that long-term vagal electrical stimulation could reduce food intake and body weight in rats [79]. Therefore, in addition to the release of 5-HT, CCK, and GLP-1, other mechanisms of action, including the direct stimulation of the vagal afferent nerves by saponins, should be explored.

Based on our experimental evidence, various health/functional foods and beverages prepared from tea-flower have been recently developed in Japan. Recent clinical studies have demonstrated that food comprising the tea-flower extract could effectively reduce postprandial blood triglyceride levels and body fat [80, 81].

Regarding anti-obesity effects of the extract highly containing oleanolic acid glycosides, Han et al. reported that the ethanol extract of *K. scoparia* fruit prevented obesity induced by a high-fat diet for 9 weeks in mice. Briefly, the ethanol extract of *K. scoparia* fruit prevented the increases in body weight and parametrial adipose tissue weight induced by the high-fat diet. Furthermore, consumption of a high-fat diet containing 1% or 3% extract significantly increased the fecal content and the fecal triglyceride levels at day 3. The ethanol extract (250 mg/kg/day) and total saponins (100 mg/kg/day) of *K. scoparia* inhibited increased plasma triglyceride levels 2 or 3 h after the oral administration of the lipid emulsion. Total saponins, momordin Ic (**14**), 2′-*O*-β-d-glucopyranosyl momordin Ic and 2′-*O*-β-d-glucopyranosyl momordin IIc inhibited the pancreatic lipase activity (in vitro). They concluded that the anti-obesity actions of *K. scoparia* extract in mice fed a high-fat diet might be partly mediated through delaying the intestinal absorption of dietary fat by inhibiting pancreatic lipase activity [82].

It should be noted that the gastrointestinal tract is an important action site of saponins, with rapid action observed before absorption after hydrolysis by intestinal bacteria; however, further pharmacological effects of various sapogenols of saponins should be investigated. As described in the reports that several flavonoid glycosides, but not aglycone, and certain carbohydrate chains in polysaccharides activated the immunity in the intestinal tract [83, 84], the role of the glycosides including saponins in the gastrointestinal tract should be carefully considered.

## References

[1] Li F, Liu B, Li T, Wu Q, Xu Z, Gu Y, Li W, Wang P, Ma T, Lei H (2020). Review of constituents and biological activities of triterpene saponins from Glycyrrhizae Radix et Rhizoma and its solubilization characteristics. Molecules.

[2] Shi Z-Y, Zeng J-Z, Wong AST (2019). Chemical structures and pharmacological profiles of ginseng saponins. Molecules.

[3] Li X, Li X, Huang N, Liu R, Sun R (2018). A comprehensive review and perspectives on pharmacology and toxicology of saikosaponins. Phytomedicine.

[4] Matsuda H (2021). Research for new biofunctional effects of triterpene saponins. Bull Kyoto Pharm Univ.

[5] Yoshikawa M, Murakami T, Harada E, Murakami N, Yamahara J, Matsuda H (1996). Bioactive saponins and glycosides. VI. Elatosides A and B, potent inhibitors of ethanol absorption, from the bark of *Aralia elata* Seem. (Araliaceae): the structure-requirement in oleanolic acid glucuronide-saponins for the inhibitory activity. Chem Pharm Bull.

[6] Yoshikawa M, Yoshizumi S, Ueno T, Matsuda H, Murakami T, Yamahara J, Murakami N (1995). Medicinal foodstuffs. I. Hypoglycemic constituents from a garnish foodstuff "Taranome," the young shoot of *Aralia elata* Seem.: elatosides G, H, I, J, and K. Chem Pharm Bull.

[7] Yoshikawa M, Matsuda H, Emiko E, Murakami T, Wariishi N, Yamahara J, Murakami N (1994). Elatoside E, a new hypoglycemic principle from the root cortex of *Aralia elata* Seem.: structure-related hypoglycemic activity of oleanolic acid glycosides. Chem Pharm Bull.

[8] Yoshikawa M, Murakami T, Harada E, Murakami N, Yamahara J, Matsuda H (1996). Bioactive saponins and glycosides. VII. On the hypoglycemic principles from the root cortex of *Aralia elata* Seem.: structure related hypoglycemic activity of oleanolic acid oligoglycoside. Chem Pharm Bull.

[9] Yoshikawa M, Dai Y, Shimada H, Morikawa T, Matsumura N, Yoshizumi S, Matsuda H, Matsuda H, Kubo M (1997). Studies on Kochiae Fructus. II. On the saponin constituents from the fruit of Chinese *Kochia scoparia* (Chenopodiaceae): chemical structures of kochianosides I, II, III, and IV. Chem Pharm Bull.

[10] Yoshikawa M, Shimada H, Morikawa T, Yoshizumi S, Matsumura N, Murakami T, Matsuda H, Hori K, Yamahara J (1997). Medicinal foodstuffs. VII. On the saponin constituents with glucose and alcohol absorption-inhibitory activity from a food garnish "Tonburi", the fruit of Japanese *Kochia scoparia* (L.) Schrad.: structures of scoparianosides A, B, and C. Chem Pharm Bull.

[11] Yoshikawa M, Murakami T, Ueno T, Kadoya M, Matsuda H, Yamahara J, Murakami N (1995). Bioactive saponins and glycosides. I. Senegae radix. (1): *E*-senegasaponins a and b and *Z*-senegasaponins a and b, their inhibitory effect on alcohol absorption and hypoglycemic activity. Chem Pharm Bull.

[12] Yoshikawa M, Murakami T, Matsuda H, Ueno T, Kadoya M, Yamahara J, Murakami N (1996). Bioactive saponins and glycosides. II. Senegae Radix. (2): chemical structures, hypoglycemic activity, and ethanol absorption-inhibitory effect of * E * -Senegasaponin c, * Z * -senegasaponin c, and * Z * -senegins II, III, and IV. Chem Pharm Bull.

[13] Yoshikawa M, Murakami T, Yoshizumi S, Murakami N, Yamahara J, Matsuda H (1996). Bioactive saponins and glycosides. V. Acylated polyhydroxyolean-12-ene triterpene triterpene oligoglycosides, camelliasaponins A_1_, A_2_, B_1_, B_2_, C_1_, and C_2_, from the seeds of *Camellia japonica* L.: structures and inhibitory activity on alcohol absorption. Chem Pharm Bull.

[14] Yoshikawa M, Murakami T, Matsuda H, Yamahara J, Murakami N, Kitagawa I (1996). Bioactive saponins and glycosides. III. Horse chestnut. (1): The structures, inhibitory effects on ethanol absorption, and hypoglycemic activity of escins Ia, Ib, IIa, IIb, and IIIa from the seeds of *Aesculus hippocastanum* L. Chem Pharm Bull.

[15] Yoshikawa M, Murakami T, Yamahara J, Matsuda H (1998). Bioactive saponins and glycosides. XII. Horse chestnut. (2): structures of escins IIIb, IV, V, and VI and isoescins Ia, Ib, and V, acylated polyhydroxyoleanene triterpene oligoglycosides, from the seeds of horse chestnut tree (*Aesculus hippocastanum* L., Hippocastanaceae). Chem Pharm Bull.

[16] Yoshikawa M, Murakami T, Kishi A, Kageura T, Matsuda H (2001). Medicinal flowers. III. Marigold. (1): hypoglycemic, gastric emptying inhibitory, and gastroprotective principles and new oleanane-type triterpene oligoglycosides, calendasaponins A, B, C, and D, from Egyptian *Calendula officinalis*. Chem Pharm Bull.

[17] Yoshikawa M, Murakami T, Kadoya M, Matsuda H, Yamahara J, Muraoka O, Murakami N (1995). Betavulgarosides I, II, III, IV, and V, hypoglycemic glucuronide saponins from the roots and leaves of *Beta vulgaris* L. (sugar beet). Heterocycles.

[18] Yoshikawa M, Murakami T, Kadoya M, Matsuda H, Muraoka O, Yamahara J, Murakami N (1996). Medicinal foodstuffs. III. Sugar Beet. (1): hypoglycemic oleanolic acid oligoglycosides, betavulgarosides I, II, III, and IV, from the root of *Beta vulgaris* L. (Chenopodiaceae). Chem Pharm Bull.

[19] Yoshikawa M, Murakami T, Kodoya M, Yamahara J, Matsuda H (1998). Medicinal foodstuffs. XV. Sugar beet. (2): structures of betavulgarosides V, VI, VII, VIII, IX, and X from the roots and leaves of sugar beet (*Beta vulgaris* L., Chenopodiaceae). Chem Pharm Bull.

[20] Yoshikawa M, Murakami T, Kadoya M, Li Y, Murakami N, Yamahara J, Matsuda H (1997). Medicinal foodstuffs. IX. The inhibitors of glucose absorption from the leaves of *Gymnema sylvestre* R. Br. (Asclepiadaceae): structures of gymnemosides a and b. Chem Pharm Bull.

[21] Yoshikawa M, Murakami T, Matsuda H (1997). Medicinal foodstuffs. X. Structures of new triterpene glycosides, gymnemosides-c, -d, -e, and -f, from the leaves of *Gymnema sylvestre* R. Br.: influence of Gymnema glycosides on glucose uptake in rat small intestinal fragments. Chem Pharm Bull.

[22] Matsuda H, Li Y, Murakami T, Matsumura N, Yamahara J, Yoshikawa M (1998). Antidiabetic principles of natural medicines. III. Structure-related inhibitory activity and action mode of oleanolic acid glycosides on hypoglycemic activity. Chem Pharm Bull.

[23] Matsuda H, Murakami T, Li Y, Yamahara J, Yoshikawa M (1998). Mode of action of escins Ia and IIa and *E, *Z-senegin II on glucose absorption in gastrointestinal tract. Bioorg Med Chem.

[24] Matsuda H, Li Y, Murakami T, Yamahara J, Yoshikawa M (1999). Structure-related inhibitory activity of oleanolic acid glycosides on gastric emptying in mice. Bioorg Med Chem.

[25] Young JB, Einhorn D, Landsberg L (1983). Decreased sympathetic (SNS) activity in interscapular brown adipose tissue (IBAT) of streptozotocin-treated rats. Diabetes.

[26] Yoshida T, Nishioka H, Nakamura Y, Kondo M (1985). Reduced noradrenaline turnover in streptozotocin-induced diabetic rats. Diabetologia.

[27] Matsuda H, Li Y, Yamahara J, Yoshikawa M (1999). Inhibition of gastric emptying by triterpene saponin, momordin Ic, in mice: roles of blood glucose, capsaicin-sensitive sensory nerves, and central nervous system. J Pharmacol Exp Ther.

[28] Young A (2005). Inhibition of gastric emptying. Adv Pharmacol.

[29] Matsuda H, Li Y, Murakami T, Yamahara J, Yoshikawa M (1999). Effects of escins Ia, Ib, IIa, and IIb from horse chestnuts on gastric emptying in mice. Eur J Pharmacol.

[30] Matsuda H, Li Y, Yoshikawa M (2000). Roles of endogenous prostaglandins and nitric oxide in inhibitions of gastric emptying and accelerations of gastrointestinal transit by escins Ia, Ib, IIa, and lIb in mice. Life Sci.

[31] Matsuda H, Li Y, Yoshikawa M (2000). Possible involvement of dopamine and dopamine_2_ receptors in the inhibitions of gastric emptying by escin Ib in mice. Life Sci.

[32] Namiki T, Hoshino T, Egashira N, Kogure T, Endo M, Homma M (2022). A review of frequently used Kampo prescriptions part 1. Daikenchuto. Tradit Kampo Med.

[33] Li Y, Matsuda H, Yoshikawa M (1999). Effects of oleanolic acid glycosides on gastrointestinal transit and ileus in mice. Bioorg Med Chem.

[34] Matsuda H, Li Y, Yoshikawa M (1999). Effects of escins Ia, Ib, IIa, and IIb from horse chestnuts on gastrointestinal transit and ileus in mice. Bioorg Med Chem.

[35] Matsuda H, Li Y, Yoshikawa M (2000). Possible involvement of 5-HT and 5-HT_2_ receptors in acceleration of gastrointestinal transit by escin Ib in mice. Life Sci.

[36] Matsuda H, Li Y, Murakami T, Yamahara J, Yoshikawa M (1998). Protective effects of oleanolic acid oligoglycosides on ethanol- or indomethacin-induced gastric mucosal lesions in rats. Life Sci.

[37] Matsuda H, Li Y, Yoshikawa M (1999). Gastroprotections of escins Ia, Ib, IIa, and IIb on ethanol-induced gastric mucosal lesions in rats. Eur J Pharmacol.

[38] Suzabo S (2014). "Gastric cytoprotection" is still relevant. Gastroenterol Hepatol.

[39] Matsuda H, Li Y, Yoshikawa M (1999). Roles of capsaicin-sensitive sensory nerves, endogenous nitric oxide, sulfhydryls, and prostaglandins in gastroprotection by momordin Ic, an oleanolic acid oligoglycoside, on ethanol-induced gastric mucosal lesions in rats. Life Sci.

[40] Matsuda H, Dai Y, Ido Y, Ko S, Yoshikawa M, Kubo M (1997). Studies on Kochiae Fructus III. Antinociceptive and antiinflammatory effects of 70% ethanol extract and its component, momordin Ic from dried fruits of *Kochia scoparia* L. Biol Pharm Bull.

[41] Matsuda H, Dai Y, Ido Y, Murakami T, Matsuda H, Yoshikawa M, Kubo M (1998). Studies on Kochiae Fructus. V. Antipruritic effects of oleanolic acid glycosides and the structure-requirement. Biol Pharm Bull.

[42] Gallelli L (2019). Escin: a review of its anti-edematous, anti-inflammatory, and venotonic properties. Drug Des Devel Ther.

[43] Matsuda H, Li Y, Murakami T, Ninomiya K, Yamahara J, Yoshikawa M (1997). Effects of escins Ia, Ib, IIa, and IIb from horse chestnut, the seeds of *Aesculus hippocastanum* L., on acute inflammation in animals. Biol Pharm Bull.

[44] Goyal RK, Guo Y, Mashimo H (2019). Advances in the physiology of gastric emptying. Neurogastroenterol Motil.

[45] Gershon MD (2013). 5-Hydroxytryptamine (serotonin) in the gastrointestinal tract. Curr Opin Endocrinol Diabetes Obes.

[46] Khan N, Mukhtar H (2018). Tea polyphenols in promotion of human health. Nutrients.

[47] Tang G-Y, Meng X, Gan R-Y, Zhao C-N, Liu Q, Feng Y-B, Li S, Wei X-L, Atanasov AG, Corke H, Li H-B (2019). Health functions and related molecular mechanisms of tea components: an update review. Int J Mol Sci.

[48] Yang C-S, Zhang J, Zhang L, Huang J, Wang Y (2016). Mechanisms of body weight reduction and metabolic syndrome allevation by tea. Mol Nutr Food Res.

[49] Kitagawa I, Hori K, Motozawa T, Murakami T, Yoshikawa M (1998). Structures of new acylated oleanene-type triterpene oligoglycosides, theasaponins E_1_ and E_2_, from the seeds of tea plant, *Camellia sinensis* (L.) O. Kuntze Chem Pharm Bull.

[50] Murakami T, Nakamura J, Matsuda H, Yoshikawa M (1999). Bioactive saponins and glycosides. XV. Saponin constituents with gastroprotective effect from the seeds of tea plant, *Camellia sinensis* L. var. *assamica* Pierre, cultivated in Sri Lanka: structures of assamsaponins A, B, C, D, and E. Chem Pharm Bull.

[51] Murakami T, Nakamura J, Kageura T, Matsuda H, Yoshikawa M (2000). Bioactive saponins and glycosides. XVII. Inhibitory effect on gastric emptying and accelerating effect on gastrointestinal transit of tea saponins: structures of assamsaponins F, G, H, I, and J from the seeds and leaves of the tea plant. Chem Pharm Bull.

[52] Yoshikawa M, Morikawa T, Li N, Nagatomo A, Li X, Matsuda H (2005). Bioactive saponins and glycosides. XXIII. Triterpene saponins with gastroprotective effect from the seeds of *Camellia sinensis* –theasaponins E_3_, E_4_, E_5_, E_6_, and E_7_–. Chem Pharm Bull.

[53] Morikawa T, Li N, Nagatomo A, Matsuda H, Li X, Yoshikawa M (2006). Triterpene saponins with gastroprotective effects from tea seed (the seeds of *Camellia sinensis*). J Nat Prod.

[54] Morikawa T, Matsuda H, Li N, Nakamura S, Li X, Yoshikawa M (2006). Bioactive saponins and glycosides. XXVI. New triterpene saponins, theasaponins E_10_, E_11_, E_12_, E_13_, and G_2_, from the seeds of tea plant (*Camellia sinensis*). Heterocycles.

[55] Yoshikawa M, Morikawa T, Nakamura S, Li N, Li X, Matsuda H (2007). Bioactive saponins and glycosides. XXV. Acylated oleanane-type triterpene saponins from the seeds of tea plant (*Camellia sinensis*). Chem Pharm Bull.

[56] Morikawa T, Matsuda H, Li N, Li X, Yoshikawa M (2007). Bioactive saponins and glycosides part 29. Acylated oleanane-typetriterpene saponins: theasaponins A_6_, A_7_, and B_5_ from the seeds of *Camellia sinensis*. Helv Chim Acta.

[57] Morikawa T, Nakamura S, Kato Y, Muraoka O, Matsuda H, Yoshikawa M (2007). Bioactive saponins and glycosides. XXVIII. New triterpene saponins, foliatheasaponins I, II, III, IV, and V, from Tencha (the leaves of *Camellia sinensis*). Chem Pharm Bull.

[58] Yoshikawa M, Morikawa T, Yamamoto K, Kato Y, Nagatomo A, Matsuda H (2005). Floratheasaponins A-C, acylated oleanane-type triterpene oligoglycosides with anti-hyperlipidemic activities from flowers of tea plant (*Camellia sinensis*). J Nat Prod.

[59] Matsuda H, Hamao M, Nakamura S, Kon'i H, Murata M, Yoshikawa M (2012). Medicinal flowers. XXXIII. Anti-hyperlipidemic and anti-hyperglycemic effects of chakasaponins I-III and structure of chakasaponin IV from flower buds of Chinese tea plant (*Camellia sinensis*). Chem Pharm Bull.

[60] Yoshikawa M, Wang T, Sugimoto S, Nakamura S, Nagatomo A, Matsuda H, Harima S (2008). Functional saponins in tea flower (flower buds of *Camellia sinensis*): gastroprotective and hypoglycemic effects of floratheasaponins and qualitative and quantitative analysis using HPLC. Yakugaku Zasshi.

[61] Hamao M, Matsuda H, Nakamura S, Nakashima S, Semura S, Maekubo S, Wakasugi S, Yoshikawa M (2011). Anti-obesity effects of the methanolic extract and chakasaponins from the flower buds of *Camellia sinensis* in mice. Bioorg Med Chem.

[62] Yoshikawa M, Sugimoto S, Kato Y, Nakamura S, Wang T, Yamashita C, Matsuda H (2009). Acylated oleanane-type triterpene saponins with acceleration of gastrointestinal transit and inhibitory effect on pancreatic lipase from flower buds of Chinese tea plant (*Camellia sinensis*). Chem Biodiv.

[63] Yoshikawa M, Nakamura S, Kato Y, Matsuhira K, Matsuda H (2007). Medicinal flowers. XIV. New acylated oleanane-type triterpene oligoglycosides with antiallergic activity from flower buds of Chinese tea plant (*Camellia sinensis*). Chem Pharm Bull.

[64] Ohta T, Nakamura S, Nakashima S, Matsumoto T, Ogawa K, Fujimoto K, Fukaya M, Yoshikawa M, Matsuda H (2015). Acylated oleanane-type triterpene oligoglycosides from the flower buds of *Camellia sinensis* var. *assamica*. Tetrahedron.

[65] Sugimoto S, Yoshikawa M, Nakamura S, Matsuda H (2009). Medicinal flowers. XXV. Structures of floratheasaponin J and chakanoside II from Japanese tea flower, flower buds of *Camellia sinensis*. Heterocycles.

[66] Yoshikawa M, Sugimoto S, Nakamura S, Matsuda H (2008). Medicinal flowers. XXII. Structures of chakasaponins V and VI, chakanoside I, and chakaflavonoside A from flower buds of Chinese tea plant (*Camellia sinensis*). Chem Pharm Bull.

[67] Morikawa T, Miyake S, Miki Y, Ninomiya K, Yoshikawa M, Muraoka O (2012). Quantitative analysis of acylated oleanane-type triterpene saponins, chakasaponins I-III and floratheasaponins A–F, in the flower buds of *Camellia sinensis* from different regional origins. J Nat Med.

[68] Morikawa T, Ninomiya K, Miyake S, Miki Y, Okamoto M, Yoshikawa M, Muraoka O (2013). Flavonol glycosides with lipid accumulation inhibitory activity and simultaneous quantitative analysis of 15 polyphenols and caffeine in the flower buds of *Camellia sinensis* from different regions by LCMS. Food Chem.

[69] Morikawa T, Lee IJ, Okugawa S, Miyake S, Miki Y, Ninomiya K, Kitagawa N, Yoshikawa M, Muraoka O (2013). Quantitative analysis of catechin, flavonoid, and saponin constituents in "tea flower", the flower buds of *Camellia sinensis*, from different regions in Taiwan. Nat Prod Commun.

[70] Shimpson KA, Martin NM, Bloom SR (2009). Hypothalamic regulation of food intake and clinical therapeutic applications. Arq Bras Endocrinol Metab.

[71] Takeda H, Sadakane C, Hattori T, Katsurada T, Ohkawara T, Nagai K, Asaka M (2008). Rikkunshito, an herbal medicine, suppresses cisplatin-induced anorexia in rats *via* 5-HT_2_ receptor antagonism. Gastroenterology.

[72] Yakabi K, Kurosawa S, Tamai M, Yuzurihara M, Nahata M, Ohno S, Ro S, Kato S, Aoyama T, Sakurada T, Takabayashi H, Hattori T (2010). Rikkunshito and 5-HT_2C_ receptor antagonist improve cisplatin-induced anorexia *via* hypothalamic ghrelin interaction. Regul Pept.

[73] Hattori T (2010). Rikkunshito and ghrelin. Int J Pept.

[74] Henschler D, Hempel K, Schultze B, Maurer W (1971). The pharmakokinetics of escin. Arzneimittelforschung.

[75] Côté CD, Zadeh-Tahmasebi M, Rasmussen BA, Duca FA, Lam TKT (2014). Hormonal signaling in the gut. J Biol Chem.

[76] McCann MJ, Verbalis JG, Stricker EM (1989). LiCl and CCK inhibit gastric emptying and feeding and stimulate OT secretion in rats. Am J Physiol.

[77] Zhang T, Perkins MH, Chang H, Han W, de Araujo IE (2022). An inter-organ neural circuit for appetite suppression. Cell.

[78] Tominaga K, Kido T, Ochi M, Sadakane C, Mase A, Okazaki H, Yamagami H, Tanigawa T, Watanabe K, Watanabe T, Fujiwara Y, Oshitani N, Arakawa T (2011). Traditional Japanese medicine rikkunshito promotes gastric emptying *via* the antagonistic action of the 5-HT_3_ receptor pathway in rats. Evid Based Complement Alternat Med.

[79] Bugajski AJ, Gil K, Ziomber A, Zurowski D, Zaraska W, Thor PJ (2007). Effect of long-term vagal stimulation on food intake and body weight during diet induced obesity in rats. J Physiol Pharmacol.

[80] Takeda K, Nakamura T, Shimoo T, Matuura Y, Kondo S, Takeda R (2021). Effects of tea flower extract containing foods on postprandial elevations triglycerides –Randomized, double-blind, pracebo-controlled, crossover study. Jpn Pharmacol Ther.

[81] Nakamura T, Natsume R, Matsuura Y, Kondo S, Takeda R (2023). Effects of ingesting food containing tea flower extract with standardized chakasaponin on the reduction of human body fat—a randomized, double-blind, pracebo-controlled, parallel-group study. Jpn Pharmacol Ther.

[82] Han L-K, Nose R, Li W, Gong X-J, Zheng Y-N, Yoshikawa M, Koike K, Nikaido T, Okuda H, Kimura Y (2006). Reduction of fat storage in mice fed a high-fat diet long term by treatment with saponins prepared from *Kochia scoparia* fruit. Phytother Res.

[83] Yonekawa M, Shimizu M, Kaneko A, Matsumura J, Takahashi H (2019). Suppression of R5-type of HIV-1 in CD4^+^ NKT cells by Vδ1^+^ T cells activated by flavonoid glycosides, hesperidin and linarin. Sci Rep.

[84] Kiyohara H, Matsuzaki T, Matsumoto T, Nagai T, Yamada H (2008). Elucidation of structures and functions through Peyer's patches of responsible carbohydrate chains in intestinal Immune system modulating polysaccharides from Japanese medicinal herbs. Yakugaku Zasshi.

[85] Matsuda H, Nakamura S, Morikawa T, Muraoka O, Yoshikawa M (2016). New biofunctional effects of flower buds of *Camellia sinensis* and its bioactive acylated oleanane-type triterpene oligoglycosides. J Nat Med.

