# Publisher Correction: New biofunctional effects of oleanane-type triterpene saponins

**DOI:** 10.1007/s11418-023-01736-4

**Published:** 2023-08-03

**Authors:** Hisashi Matsuda, Toshio Morikawa, Seikou Nakamura, Osamu Muraoka, Masayuki Yoshikawa

**Affiliations:** 1 Department of Pharmacognosy, Kyoto Pharmaceutical University, Misasagi, Yamashina-Ku, Kyoto, 607-8412 Japan; 2 Pharmaceutical Research and Technology Institute, Kindai University, 3-4-1 Kowakae, Higashi-Osaka, Osaka, 577-8502 Japan

**Correction to: Journal of Natural Medicines** 10.1007/s11418-023-01730-w

In the original version of this article published on 12 July 2023, Fig. 4 contained an error due to a mistake while typesetting. The number labels in Fig. 4 1), which shows the chemical structure of olean-12-en-28-oic acid 3-*O*-monodesmoside, are incorrect. The incorrect “oleanolic acid 3-*O*-glucuronide (**18**)” has now been replaced with the correct “oleanolic acid 3-*O*-glucuronide (**16**)”, and the incorrect “momordin lc (**16**)” has now been replaced with the correct “momordin lc (**14**)”.

**Fig. 4:**
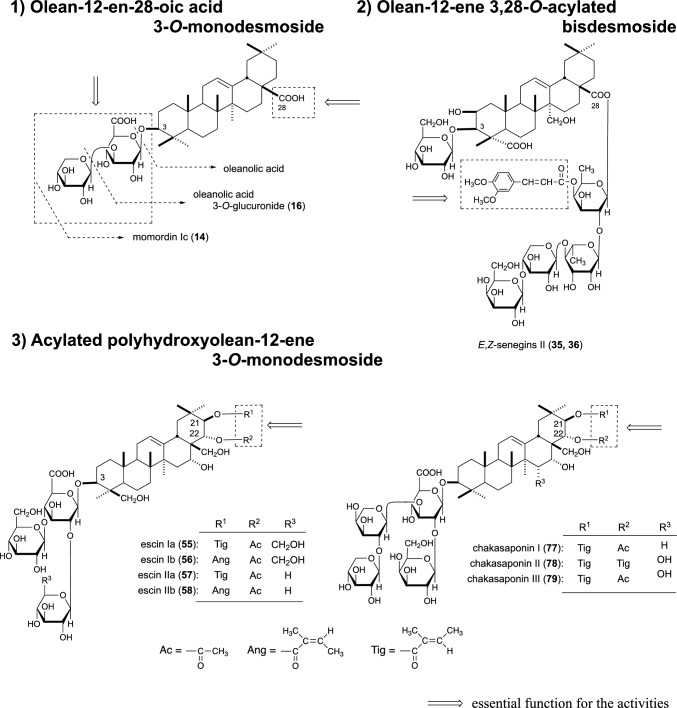
Three type of the active oleanane-type triterpene saponins

The original article has been corrected. The publisher regrets the error.

